# Trait, state, and behavior in adolescent NSSI addiction: a serial mediation model linking childhood trauma to NSSI addiction

**DOI:** 10.3389/fpsyt.2026.1942023

**Published:** 2026-09-28

**Authors:** Lan Hu, Feiyan Wang, Li Pu, Guojian Yan, Yunge Li

**Affiliations:** The Clinical Hospital of Chengdu Brain Science Institute, MOE Key Lab for Neuroinformation, University of Electronic Science and Technology of China, Chengdu, China

**Keywords:** addictive behavior, childhood trauma, depression, impulsivity, nonsuicidal self-injurious behavior

## Abstract

**Background:**

Despite growing recognition of non-suicidal self-injury (NSSI) as a potential behavioral addiction, the sequential mechanisms linking childhood trauma to NSSI addiction remain unclear. This study examined a serial mediation model testing whether trait impulsivity and depressive symptoms sequentially mediate this relationship, with NSSI function as a behavioral coping mechanism.

**Methods:**

This cross-sectional study included 163 Chinese adolescents diagnosed with major depressive disorder and a history of NSSI. Participants completed the Childhood Trauma Questionnaire-Short Form, the Short UPPS-P Impulsive Behavior Scale, the Children’s Depression Inventory, and the Ottawa Self-Injury Inventory (Functions and Addictive Features subscales). Serial mediation analysis (PROCESS Model 6, 5,000 bootstrap resamples), controlling for demographic and clinical covariates, tested the hypothesized five-step sequential model.

**Results:**

Childhood trauma was positively associated with NSSI addiction, and this association was predominantly mediated: the total indirect effect accounted for 56.2% of the total effect. Indirect effects involving NSSI function in combination with upstream mediators were significant, including the full sequential path (B = 0.0033, 95% CI [0.0007, 0.0083]), whereas simple mediations via impulsivity, depression, or NSSI function alone were not.

**Conclusion:**

These findings support a trait-state-behavior-addiction cascade in which childhood trauma is associated with trait impulsivity and depressive symptoms that, in combination with the reinforcing function of NSSI, are linked to addictive features of NSSI. The model advances theoretical understanding of NSSI addiction and offers actionable clinical targets for prevention and intervention. Longitudinal studies are needed to confirm causal directions.

## 1 Introduction

Non-suicidal self-injury (NSSI) refers to the deliberate, self-inflicted destruction of body tissue without suicidal intent and for purposes not socially sanctioned (1). The DSM-5 has included NSSI as a condition warranting further study, reflecting its clinical significance. NSSI is particularly prevalent during adolescence: a meta-analysis reported a pooled prevalence of 17.7% among adolescents aged 10–19 years from 17 countries, with higher rates in females (21.4%) than in males (13.7%) (2). Moreover, NSSI rarely occurs in isolation; a recent meta-analysis by Wu and colleagues (3), which included 29 studies with 12,934 depressed adolescents, estimated that the lifetime prevalence of self-injury in this population is 52%. Thus, the co-occurrence of NSSI and depression is not rare but rather a pervasive clinical reality. Despite growing awareness, the underlying mechanisms of NSSI in depressed adolescents remain incompletely understood, highlighting the need for continued research into its etiology and maintenance factors.

In the last decade, researchers have proposed that NSSI, particularly repetitive NSSI, can be conceptualized as a behavioral addiction, sharing similar phenomenological features with substance use disorders, including craving, tolerance, withdrawal, loss of control, and continued engagement despite negative consequences (4, 5). Neurobiological evidence supports this addictive model of NSSI, revealing findings similar to those observed in addictive disorders. NSSI is thought to trigger the release of endogenous opioids, providing pain relief and reinforcing repetition (6). Additionally, neuroimaging studies show reward circuitry involvement: increased putamen activation to monetary gains (7), and reduced nucleus accumbens activation during punishment avoidance compared to reward selection (8). A resting-state fMRI study directly comparing adolescents with addictive vs. non-addictive NSSI found functional connectivity alterations in reward and emotion regulation circuits (9). However, despite growing recognition of NSSI as a potential behavioral addiction, the specific mechanisms through which distal risk factors sequentially contribute to the development of NSSI addiction remain incompletely understood.

It is important to note that NSSI addiction is not currently recognized as a formal diagnostic entity in DSM-5 or ICD-11; NSSI is listed in DSM-5 only as a condition warranting further study. Accordingly, in the present study, the term “NSSI addiction” refers to addiction-like features (e.g., craving, tolerance, withdrawal, and loss of control) measured by the Addictive Features subscale of the Ottawa Self-Injury Inventory (OSI), rather than to a formal clinical diagnosis. This construct-level caution is maintained throughout.

The trait-state model, originally developed for anxiety (10), provides a framework for understanding how enduring dispositions (traits) and transient emotional conditions (states) jointly influence addictive behaviors. Traits create a general vulnerability, while states trigger symptom manifestation. This model has been applied to understand the development of addictive behaviors. For example, distinct impulsivity facets have been linked to different pathways in alcohol use disorder: ‘lack of premeditation’ predicted negative consequences through heavy drinking, while ‘negative urgency’ predicted consequences through heightened depression and stress (11). Demetrovics and colleagues (12), in a large-scale study of various behavioral addictions, found that while early-stage addiction is primarily driven by reward-seeking impulsivity (trait), more severe cases are characterized by relief-driven compulsivity (state), which maintains the addictive cycle. This temporal shift from impulsivity to compulsivity suggests that an initial vulnerability (trait) can, under the influence of negative reinforcement mechanisms (state), evolve into a compulsive behavior pattern.

Prior research has also provided empirical grounding for applying the trait-state framework to understand NSSI addiction. Among trait-level factors, impulsivity has emerged as one of the most robust and consistently documented risk factors for NSSI (13), and is a shared feature across different addictive disorders or conditions, including alcohol use (14), cigarette smoking (15), and gaming disorder (16). Impulsivity refers to a predisposition toward rapid, unplanned reactions to internal or external stimuli without regard to the negative consequences of these reactions for the impulsive individual or for others (17). Among various dimensions of impulsivity, Negative Urgency and Lack of Premeditation have emerged as the most critical traits differentiating individuals with NSSI from those without (18, 19). More importantly, these impulsivity traits also interact with emotional states (e.g., sadness and criticism) to amplify NSSI severity (20, 21). Given that addictive features represent a particularly severe consequence of repetitive NSSI, identifying their determinants is of clinical importance. Yet, no study has investigated the role of trait impulsivity in NSSI addiction, nor the sequential relationship between impulsivity and emotional states within a trait-state framework.

A growing body of evidence has established a robust and consistent link between childhood trauma and the development of trait impulsivity. A meta-analysis of 55 studies found a positive association between childhood maltreatment and general trait impulsivity (22). Longitudinal data from the Adolescent Brain Cognitive Development study showed that early trauma predicts later negative and positive urgency (23). Impulsivity also mediates the pathway from childhood trauma to depressive outcomes. Among hospitalized adolescents with NSSI, childhood maltreatment was associated with higher impulsivity, which in turn predicted more severe depression and anxiety (24). Similar mediation effects have been reported in incarcerated youth (25) and university students (26).

For depressed adolescents, NSSI often serves as a maladaptive coping strategy, primarily for automatic negative reinforcement, specifically affect regulation—the use of self-injury to down-regulate aversive internal states—followed by anti-dissociation and self-punishment (27, 28). Repeated engagement in NSSI for these functions may drive the transition to an addictive pattern characterized by craving, tolerance, withdrawal, and loss of control (29). Critically, because these reinforcing functions are engaged in response to aversive affective states and enacted with little forethought, NSSI function is expected to operate downstream of impulsivity and depression rather than as an independent mediator. The OSI assesses both functional motivations and addictive features, making it a valuable tool for this research (30, 31). The OSI-Functions subscale assesses the functional motivations underlying NSSI behavior, with higher scores indicating a greater tendency to rely on self-injury as a maladaptive coping strategy, while the OSI-Addictive Features subscale evaluates addictive characteristics, with higher scores reflecting more severe self-injurious behavior with potential addictive features.

Recent studies have further elucidated mediating mechanisms in the trauma–NSSI pathway. Shi et al. (32) found that alexithymia and NSSI function serially mediated the relationship between childhood trauma and NSSI, highlighting the role of emotion regulation deficits and the functional reinforcement of self-injury. Yao et al. (33) demonstrated that childhood trauma increases NSSI risk through a chain mediation of reduced social support and increased depression, underscoring the importance of environmental and emotional pathways. These studies establish childhood trauma as a distal risk factor for NSSI and support the idea of sequential cascades, but they have not examined the addictive features of NSSI as an outcome, nor integrated trait impulsivity as a proximal vulnerability.

Although impulsivity and depression are likely transactionally related over time, we place trait impulsivity upstream on the basis of (a) its conceptualization as a relatively stable disposition that precedes transient mood states (34) and (*b*) evidence that trait impulsivity prospectively predicts subsequent increases in depressive symptoms via emotion dysregulation (35). We emphasize that, given the cross-sectional design, the sequence below represents a theoretically plausible ordering rather than an empirically demonstrated causal chain. Accordingly, we propose a sequential cascade: childhood trauma (distal risk) → trait impulsivity (proximal trait) → depression (emotional state) → NSSI function (behavioral coping) → NSSI addiction (addictive outcome). We hypothesize that: (1) childhood trauma will be positively associated with NSSI addiction; (2) this association will be transmitted through the proposed sequential cascade, with trait impulsivity and depressive symptoms serving as upstream mediators and NSSI function as the final proximal mediator; and (3) only indirect pathways that pass through NSSI function will be significant, whereas truncated chains that bypass it will not. To test these hypotheses, we recruited a clinical sample of adolescents with major depressive disorder (MDD) and NSSI behavior.

## 2 Methods

### 2.1 Participants

The study protocol was approved by the Ethics Committee of the Chengdu Fourth People’s Hospital and adhered to the Declaration of Helsinki. Written informed consent was obtained from all participants (and their legal guardians for those under 18 years).

Inclusion criteria were: (1) age 12–18 years; (2) DSM-5 diagnosis of MDD; (3) history of NSSI within the past year; (4) ability to read and understand Chinese; and (5) written informed consent. NSSI was defined as deliberate, self-inflicted damage to body tissue without suicidal intent and for purposes not socially sanctioned (36). Psychiatric diagnoses and NSSI assessments were conducted by a qualified psychiatrist. Self-reported NSSI frequency in the past year was assessed using the OSI (30), with response options: 0 = none, 1 = 1–5 times, 2 = monthly, 3 = weekly, 4 = daily. Participants with a psychiatrist-confirmed NSSI history and a self-reported frequency ≥1 were included.

Exclusion criteria were: (1) any current or lifetime DSM-5 disorder other than MDD (e.g., anxiety disorders, bipolar disorder, schizophrenia); (2) a history of substance use disorder, traumatic brain injury, or loss of consciousness; and (3) intellectual disability. From December 2021 to August 2024, 168 participants were recruited from inpatient and outpatient settings at the Fourth People’s Hospital of Chengdu.

### 2.2 Demographic and clinical measures

Demographic information of the enrolled participants, including age, sex, years of education, and parental marital status, was collected. Clinical characteristics of MDD, including total illness duration (in months) and the number of episodes, were evaluated by a qualified psychiatrist. Other clinical features—namely, the frequency of NSSI over the past year, childhood trauma, impulsivity, depressive symptoms, NSSI functions, and NSSI addiction—were assessed using self-report scales. Detailed information about each scale is provided below.

#### 2.2.1 Childhoold trauma questionnaire-short form (CTQ-SF)

The Chinese version of the CTQ-SF (37, 38) was used to assess childhood maltreatment experiences. The CTQ-SF is a 28-item self-report scale (including 25 clinical items and 3 validity items) measuring five types of childhood trauma: emotional abuse, physical abuse, sexual abuse, emotional neglect, and physical neglect. Each item is rated on a 5-point Likert scale ranging from 1 (never true) to 5 (very often true). The total score ranges from 25 to 125, with higher scores indicating greater severity of childhood trauma. Cronbach’s *α* in this study was 0.889.

#### 2.2.2 Short UPPS-P impulsive behavior scale (SUPPS-P)

Trait impulsivity was measured using the SUPPS-P (39), a 20-item short form of the UPPS-P rated on a 4-point Likert scale (1 = strongly disagree to 4 = strongly agree). It assesses five distinct impulsivity dimensions: negative urgency, lack of premeditation, lack of perseverance, sensation seeking, and positive urgency. Reverse-scored items were recoded prior to computing the total score. Higher scores indicate higher levels of impulsivity. Cronbach’s *α* in this study was 0.769. Facet scores (negative urgency, positive urgency, sensation seeking, lack of perseverance, and lack of premeditation; four items each) were computed for the exploratory facet-level analyses described in Section 2.3.

#### 2.2.3 Children's depression inventory (CDI)

Depressive symptoms, representing emotional state, were assessed using the CDI (40, 41). The CDI is a 27-item self-report scale designed for children and adolescents. Each item consists of three statements scored from 0 to 2 (e.g., 0 = “I am sad once in a while”, 1 = “I am sad many times”, 2 = “I am sad all the time”), and participants are asked to select the statement that best describes their feelings over the past two weeks. Total scores range from 0 to 54, with higher scores indicating more severe depressive symptoms. Cronbach’s *α* in this study was 0.808.

#### 2.2.4 Ottawa self-injury inventory

NSSI behavior was assessed using the OSI which is a comprehensive self-report measure designed to evaluate multiple dimensions of NSSI behavior, including frequency, functions, and addictive features of the behavior (30, 42). In this study, the OSI was used to assess three aspects of NSSI.

##### 2.2.4.1 Frequency of NSSI over the past year

Frequency of NSSI over the past year was rated as described in Section 2.1.

##### 2.2.4.2 NSSI function (past month)

NSSI function over the past month was assessed using the OSI Functions subscale, where participants rated reasons for NSSI (e.g., “to relieve unbearable tension”) on a 5-point scale (0 = never a reason to 4 = always a reason). Higher scores indicate stronger endorsement of NSSI as a functional coping strategy. Cronbach’s *α* in this study was 0.944.

##### 2.2.4.3 NSSI addiction

The OSI Addictive Features subscale was used to assess addiction-like characteristics of NSSI behavior. This subscale consists of seven items, each rated on a 5-point scale from 0 (never) to 4 (always). Higher scores on this subscale indicate greater levels of NSSI addiction severity. Cronbach’s *α* in this study was 0.839.

### 2.3 Statistical analysis

Of 168 recruited participants, five were excluded due to incomplete data, yielding a final sample of 163 adolescents. Gender, parental marital status, and NSSI frequency were treated as categorical variables; all others as continuous. Analyses were performed using SPSS 27.0 (IBM Corp., Armonk, NY, USA).

#### 2.3.1 Common method bias test

Harman’s single-factor test (43) was conducted by entering all items from the CTQ-SF, SUPPS-P, CDI, OSI Functions, and OSI Addictive Features subscales into an unrotated factor analysis with the number of factors fixed at one. The first unrotated factor accounted for 18.99% of the total variance, well below the 40% threshold, indicating that common method bias was not a serious concern.

#### 2.3.2 Missing data handling

For each scale, if missing items did not exceed 20% of the total items, missing values were replaced with the participant’s mean score on completed items; otherwise, the total score was considered missing. In this study, the proportions of missing values for demographic variables, clinical characteristics, and scale total scores were all less than 10%. Multiple imputation (*m* = 10) using the Fully Conditional Specification (FCS) method was performed, with predictive mean matching for continuous variables and logistic regression for categorical variables (44). The imputation model included 12 variables: age, gender, years of education, parental marital status (nominal: 1 = married, 2 = divorced, 3 = other), total MDD duration, number of MDD episodes, NSSI frequency, and the total scores of the CTQ-SF, SUPPS-P, CDI, and the OSI Functions and OSI Addictive Features subscales. Parental marital status was imputed as a nominal variable under the FCS logistic model; after imputation, it was recoded into two dummy variables (divorced vs. married; other vs. married) for the hierarchical regression and serial mediation analyses. The first imputed dataset (Imputation_1) was used for primary analyses and for all descriptive statistics reported below; Imputation_5 and Imputation_10 were used for robustness checks (**Supplementary Materials**). For the exploratory facet-level analyses, multiple imputation was re-run with the five SUPPS-P facet scores added to the imputation model, and these analyses used the facet-inclusive imputed datasets (**Supplementary Materials**).

#### 2.3.3 Descriptive statistics

Descriptive statistics were computed for all variables using the first imputed dataset (Imputation_1, *N* = 163). Continuous variables were summarized as means (*M*), standard deviations (*SD*), medians, minimum and maximum values, skewness, and kurtosis. Categorical variables were summarized as frequencies (*n*) and percentages (%), including parental marital status (married, divorced, or other) and NSSI frequency.

#### 2.3.4 Correlation analysis

The Shapiro-Wilk test was used to assess the normality of continuous variables. Results indicated that CTQ-SF (*p* = 0.038), OSI Functions subscale (*p* = 0.016), and OSI Addictive Features subscale (*p* = 0.039) deviated significantly from normality, whereas CDI (*p* = 0.386) and SUPPS-P (*p* = 0.398) were normally distributed. Given the non-normal distribution of several core variables, Spearman’s rank-order correlation coefficients were computed to examine the bivariate relationships among CTQ-SF, SUPPS-P, CDI, OSI Functions subscale, and OSI Addictive Features subscale. 95% confidence intervals (CIs) for the Spearman correlations were estimated using Fisher’s z transformation with the Fieller–Hartley–Pearson standard-error approximation for rank correlations (45). All analyses were performed on the first imputed dataset (Imputation_1, *N* = 163).

#### 2.3.5 Hierarchical multiple regression analysis

Hierarchical multiple regression examined the independent predictive effects of childhood trauma, impulsivity, depressive severity, and NSSI function on NSSI addiction. Covariates (gender, age, years of education, total illness duration in months, and number of MDD episodes) were entered in Block 1, followed by parental marital status (two dummy variables, with married as the reference) in Block 2, CTQ-SF in Block 3, SUPPS-P in Block 4, CDI in Block 5, and the OSI Functions subscale in Block 6. *ΔR²* and associated *F*-change statistics evaluated incremental contributions. Multicollinearity was assessed using the variance inflation factor (*VIF*; a threshold of > 5 indicates concern; 46). Pooled results from the multiply imputed datasets (*m* = 10) are reported.

#### 2.3.6 Serial mediation analysis

The hypothesized sequential mediation model (childhood trauma → impulsivity → depression → NSSI function → NSSI addiction) was tested using the PROCESS macro (Version 5.0; 47) Model 6 for SPSS. 95% percentile bootstrap CIs for indirect effects were generated from 5,000 bootstrap resamples; an effect was considered significant if the CI did not include zero. Age, gender, years of education, total illness duration (in months), number of MDD episodes, and parental marital status (two dummy variables) were included as covariates. Primary analysis used Imputation_1; robustness checks were performed on Imputation_5 and Imputation_10 (see **Supplementary Materials**). Three sensitivity analyses are reported in the **Supplementary Materials**: (a) an alternative model in which the order of the first two mediators was reversed (childhood trauma → depression → impulsivity → NSSI function → NSSI addiction); (*b*) exploratory analyses in which the SUPPS-P total score was replaced, in turn, by each of its five impulsivity facets; and (*c*) a covariate-reduced serial mediation model that excluded parental marital status from the covariate set, to verify that the inclusion of marital status did not materially alter the chain of indirect effects.

## 3 Results

### 3.1 Sample characteristics

Of 163 adolescents with MDD and NSSI, the mean age was 14.80 years (*SD* = 1.68), with 128 females (78.5%) and 35 males (21.5%). Mean education was 8.95 years (*SD* = 1.70); 64.4% had married parents. Mean MDD duration was 19.62 months (*SD* = 15.07; median = 12.00); mean MDD episodes were 1.12 (*SD* = 0.41). NSSI frequency (past year): 31.9% (1–5 times), 24.5% (monthly), 34.4% (weekly), 9.2% (daily). Mean scores: CTQ-SF = 54.26 (*SD* = 15.01), SUPPS-P = 51.31 (*SD* = 7.92), CDI = 33.74 (*SD* = 7.32), OSI Functions = 46.80 (*SD* = 23.50), OSI Addictive Features = 13.48 (*SD* = 6.83). Details are presented in **Table 1**.

**Table 1:** Demographic and clinical characteristics of the sample (Imputation_1, *N* = 163).

Variable	Mean (*SD*)/*n* (%)	Median	Min	Max	Skewness	Kurtosis
Demographic characteristics						
Age (years)	14.80 (1.68)	15.00	12	18	0.24	-0.94
Gender (male)	35 (21.5%)	—	—	—	—	—
Years of education	8.95 (1.70)	9.00	6	13	0.26	-0.95
Parental marital status						
— Married	105 (64.4%)	—	—	—	—	—
— Divorced	50 (30.7%)	—	—	—	—	—
— Other	8 (4.9%)	—	—	—	—	—
Clinical characteristics						
Total MDD duration (months)	19.62 (15.07)	12.00	1	72	0.83	0.13
Number of MDD episodes	1.12 (0.41)	1.00	1	4	4.22	20.76
NSSI frequency (past year)						
— 1–5 times	52 (31.9%)	—	—	—	—	—
— Monthly	40 (24.5%)	—	—	—	—	—
— Weekly	56 (34.4%)	—	—	—	—	—
— Daily	15 (9.2%)	—	—	—	—	—
CTQ-SF total score	54.26 (15.01)	54.35	25	94	0.33	-0.17
SUPPS-P total score	51.31 (7.92)	51.00	24.21	71	-0.20	0.47
CDI total score	33.74 (7.32)	34.00	14.54	54	-0.12	0.15
OSI Functions subscale total score	46.80 (23.50)	45.24	0	128	0.55	0.29
OSI Addictive Features subscale total score	13.48 (6.83)	13.00	0	28	0.11	-0.71

*SD*, standard deviation; Min, minimum; Max, maximum; MDD, major depressive disorder; NSSI, non-suicidal self-injury; CTQ-SF, Childhood Trauma Questionnaire – Short Form; SUPPS-P, Short UPPS-P Impulsive Behavior Scale; CDI, Children’s Depression Inventory; OSI, Ottawa Self-Injury Inventory. All values are based on Imputation_1 of the multiple imputation (m = 10) that included parental marital status.

### 3.2 Bivariate correlations

Spearman’s correlations (**Table 2**) showed that all core variables were significantly intercorrelated (all *ps* < 0.01). The strongest correlation was between the OSI Functions and OSI Addictive Features (*r* = 0.483, 95% CI [0.352, 0.596]), and the weakest was between childhood trauma and NSSI function (*r* = 0.235, 95% CI [0.079, 0.379], *p* = 0.003).

**Table 2:** Spearman’s rank-order correlation matrix among core variables (Imputation_1, *N* = 163).

Variable	1	2	3	4	5
1. CTQ-SF	—				
2. SUPPS-P	0.263** [0.110, 0.405]	—			
3. CDI	0.395** [0.252, 0.520]	0.395** [0.253, 0.521]	—		
4. OSI Functions subscale	0.235** [0.079, 0.379]	0.408** [0.267, 0.532]	0.418** [0.278, 0.540]	—	
5. OSI Addictive Features	0.336** [0.188, 0.469]	0.407** [0.266, 0.531]	0.432** [0.294, 0.553]	0.483** [0.352, 0.596]	—

CTQ-SF, Childhood Trauma Questionnaire – Short Form; SUPPS-P, Short UPPS-P Impulsive Behavior Scale; CDI, Children’s Depression Inventory; OSI, Ottawa Self-Injury Inventory. Values are Spearman’s rank-order correlation coefficients (*r*) with 95% confidence intervals in brackets based on Fisher’s z transformation with the Fieller–Hartley–Pearson standard-error approximation for rank correlations. All correlations were significant at **p < 0.01 (two-tailed).

### 3.3 Hierarchical regression analysis

Hierarchical regression analysis (**Table 3**) revealed that the demographic covariates (Block 1) did not significantly predict NSSI addiction as a block (Δ*R*² = 0.039, *p* = 0.093), although gender was a significant individual predictor within this block (*p* = 0.016), whereas adding illness duration and number of MDD episodes (Block 2) significantly improved model fit (*ΔR²* = 0.050, *p* = 0.016). Parental marital status (Block 3) did not improve fit (*ΔR²* = 0.002, *p* = 0.815) but was retained as a covariate. Each psychological predictor block significantly improved model fit: childhood trauma (Block 4, *ΔR²* = 0.095, *p* < 0.001), impulsivity (Block 5, *ΔR²* = 0.091, *p* < 0.001), depressive symptoms (Block 6, *ΔR²* = 0.040, *p* = 0.004), and NSSI function (Block 7, *ΔR²* = 0.099, *p* < 0.001). In the final model, NSSI function emerged as the strongest predictor of NSSI addiction (*β* = 0.365, *p* < 0.001), followed by illness duration (*β* = 0.182, *p* = 0.008) and childhood trauma (*β* = 0.150, *p* = 0.042); depressive symptoms (*β* = 0.148, *p* = 0.060) and impulsivity (*β* = 0.128, *p* = 0.086) showed positive but non-significant unique effects. None of the remaining covariates (gender, age, years of education, number of MDD episodes, and parental marital status) was significant in the final model (*ps* > 0.05). The final model explained 41.6% of the variance (adjusted *R²* = 0.374), with all VIFs < 3.9, indicating no multicollinearity.

**Table 3:** Hierarchical regression analysis of factors associated with OSI Addictive Features (pooled across 10 imputations).

Model	Variables	*β*	*t*	*p*	*R*²	Δ*R*²	*p* for Δ*R*²	Adjusted *R*²	VIF
Model 1	Gender	0.194	2.409	0.016	0.039	0.039	0.093	0.021	1.078
	Age	-0.028	-0.195	0.845					3.424
	Years of education	0.019	0.132	0.895					3.336
Model 2	Gender	0.156	1.945	0.052	0.089	0.050	0.016	0.060	1.109
	Age	-0.122	-0.846	0.398					3.603
	Years of education	0.025	0.182	0.856					3.343
	Illness duration (months)	0.221	2.694	0.007					1.151
	Number of MDD episodes	0.072	0.939	0.348					1.021
Model 3	Gender	0.161	1.984	0.047	0.091	0.002	0.815	0.050	1.119
	Age	-0.122	-0.834	0.404					3.670
	Years of education	0.023	0.161	0.872					3.367
	Illness duration (months)	0.226	2.711	0.007					1.180
	Number of MDD episodes	0.075	0.968	0.333					1.030
	Parental marital status: divorced	-0.047	-0.590	0.555					1.069
	Parental marital status: other	-0.021	-0.259	0.796					1.063
Model 4	Gender	0.121	1.565	0.118	0.186	0.095	<0.001	0.144	1.135
	Age	-0.203	-1.441	0.150					3.738
	Years of education	0.087	0.651	0.515					3.411
	Illness duration (months)	0.177	2.212	0.027					1.206
	Number of MDD episodes	0.073	0.987	0.324					1.030
	Parental marital status: divorced	-0.121	-1.565	0.118					1.127
	Parental marital status: other	-0.062	-0.810	0.418					1.081
	CTQ-SF	0.330	4.221	<0.001					1.149
Model 5	Gender	0.091	1.235	0.217	0.278	0.091	<0.001	0.235	1.146
	Age	-0.127	-0.950	0.342					3.800
	Years of education	0.029	0.226	0.821					3.449
	Illness duration (months)	0.168	2.217	0.027					1.207
	Number of MDD episodes	0.036	0.510	0.610					1.045
	Parental marital status: divorced	-0.088	-1.188	0.235					1.139
	Parental marital status: other	-0.063	-0.862	0.389					1.081
	CTQ-SF	0.244	3.183	0.001					1.231
	SUPPS-P	0.320	4.336	<0.001					1.127
Model 6	Gender	0.051	0.690	0.490	0.317	0.040	0.004	0.272	1.187
	Age	-0.113	-0.861	0.389					3.806
	Years of education	0.060	0.479	0.632					3.473
	Illness duration (months)	0.172	2.325	0.020					1.208
	Number of MDD episodes	0.039	0.574	0.566					1.045
	Parental marital status: divorced	-0.071	-0.993	0.321					1.146
	Parental marital status: other	-0.054	-0.769	0.442					1.083
	CTQ-SF	0.165	2.070	0.038					1.389
	SUPPS-P	0.250	3.307	<0.001					1.250
	CDI	0.243	2.955	0.003					1.486
Model 7	Gender	0.062	0.910	0.363	0.416	0.099	<0.001	0.374	1.189
	Age	-0.126	-1.039	0.299					3.809
	Years of education	0.038	0.326	0.745					3.478
	Illness duration (months)	0.182	2.638	0.008					1.209
	Number of MDD episodes	0.062	0.967	0.334					1.051
	Parental marital status: divorced	-0.068	-1.004	0.315					1.147
	Parental marital status: other	-0.067	-1.012	0.312					1.084
	CTQ-SF	0.150	2.032	0.042					1.391
	SUPPS-P	0.128	1.716	0.086					1.403
	CDI	0.148	1.884	0.060					1.578
	OSI Functions subscale	0.365	4.934	<0.001					1.347

*N* = 163. Blocks: 1 = demographics; 2 = illness duration and number of MDD episodes; 3 = parental marital status (two dummies, married as reference); 4 = CTQ-SF; 5 = SUPPS-P; 6 = CDI; 7 = OSI Functions. *t* and *p* values are pooled estimates across the 10 imputed datasets (Rubin’s rules); standardized coefficients (*β*), *R*², Δ*R*², adjusted *R*², and VIF are arithmetic means across the 10 imputed datasets, as SPSS does not pool these statistics. *F*-change statistics are arithmetic means across the 10 imputed datasets: Model 1 ΔF = 2.18; Model 2 ΔF = 4.27; Model 3 ΔF = 0.21; Model 4 ΔF = 17.98; Model 5 ΔF = 19.31; Model 6 ΔF = 8.85; Model 7 ΔF = 25.62. *p* values for Δ*R*² are likewise arithmetic means of the per-imputation *F*-change *p* values. The fraction of missing information for all coefficients in Model 7 ranged from 0.005 to 0.047.

### 3.4 Serial mediation analysis

Serial mediation analysis (PROCESS Model 6, 5,000 bootstrap resamples, controlling for gender, age, years of education, illness duration, number of MDD episodes, and parental marital status) revealed that the total effect of CTQ-SF on NSSI addiction was significant (*c* = 0.1513, 95% CI [0.0814, 0.2212]), while the direct effect was attenuated and only marginally non-significant (*c’* = 0.0662, 95% CI [-0.0006, 0.1330], *p* = 0.052), indicating that the association was predominantly, though not entirely, mediated. The total indirect effect was 0.0851 (95% CI [0.0366, 0.1538]), accounting for 56.2% of the total effect. This pattern was robust across imputations: the total indirect effect and *Ind5*–*Ind7* remained significant in Imputation_5 and Imputation_10, while the direct effect was non-significant in Imputation_5 (*p* = 0.057) and significant in Imputation_10 (*p* = 0.031) (**Supplementary Materials**). Details are presented in **Table 4** and **Figure 1**.

**Table 4:** Serial mediation analysis of the pathway from childhood trauma to NSSI addiction (Imputation_1; 5,000 bootstrap resamples).

Path	Effect (*B*)	Boot *SE*	95% CI	*p*	Proportion mediated
Direct and total effects					
Total effect (*c*)	0.1513	0.0354	[0.0814, 0.2212]	<0.001	100%
Direct effect (*c*′)	0.0662	0.0338	[-0.0006, 0.1330]	0.052	—
Individual paths					
CTQ-SF → SUPPS-P (a_1_)	0.1496	0.0493	[0.0597, 0.2530]	<0.001	
SUPPS-P → CDI (a_2_)	0.2641	0.0611	[0.1374, 0.3753]	<0.001	
CDI → OSI Functions (a_3_)	0.8622	0.2739	[0.3330, 1.4064]	0.002	
OSI Functions → Addictive Features (*b*)	0.0981	0.0235	[0.0543, 0.1466]	<0.001	
Indirect effects					
Total indirect	0.0851	0.0299	[0.0366, 0.1538]	—	56.2% *
Ind1: CTQ-SF → SUPPS-P → Addictive Features	0.0186	0.0122	[-0.0002, 0.0477]	—	—
Ind2: CTQ-SF → CDI → Addictive Features	0.0227	0.0147	[-0.0030, 0.0545]	—	—
Ind3: CTQ-SF → OSI Functions → Addictive Features	0.0066	0.0130	[-0.0169, 0.0345]	—	—
Ind4: CTQ-SF → SUPPS-P → CDI → Addictive Features	0.0056	0.0045	[-0.0006, 0.0169]	—	—
Ind5: CTQ-SF → SUPPS-P → OSI Functions → Addictive Features	0.0148	0.0078	[0.0039, 0.0336]	—	9.8% *
Ind6: CTQ-SF → CDI → OSI Functions → Addictive Features	0.0135	0.0068	[0.0038, 0.0302]	—	8.9% *
Ind7: CTQ-SF → SUPPS-P → CDI → OSI Functions → Addictive Features	0.0033	0.0020	[0.0007, 0.0083]	—	2.2% *

*N* = 163. Covariates: gender, age, years of education, total illness duration (months), number of MDD episodes, and parental marital status (two dummy variables, married as reference). CIs for indirect effects and individual paths are 95% percentile bootstrap CIs based on 5,000 resamples; *p* values for the total and direct effects and individual paths are from ordinary least squares (OLS) *t*-tests. Proportion mediated = indirect effect/total effect; shown only for significant indirect effects (marked *). Robustness checks on Imputation_5 and Imputation_10 are reported in the **Supplementary Materials**.

**Figure 1:**
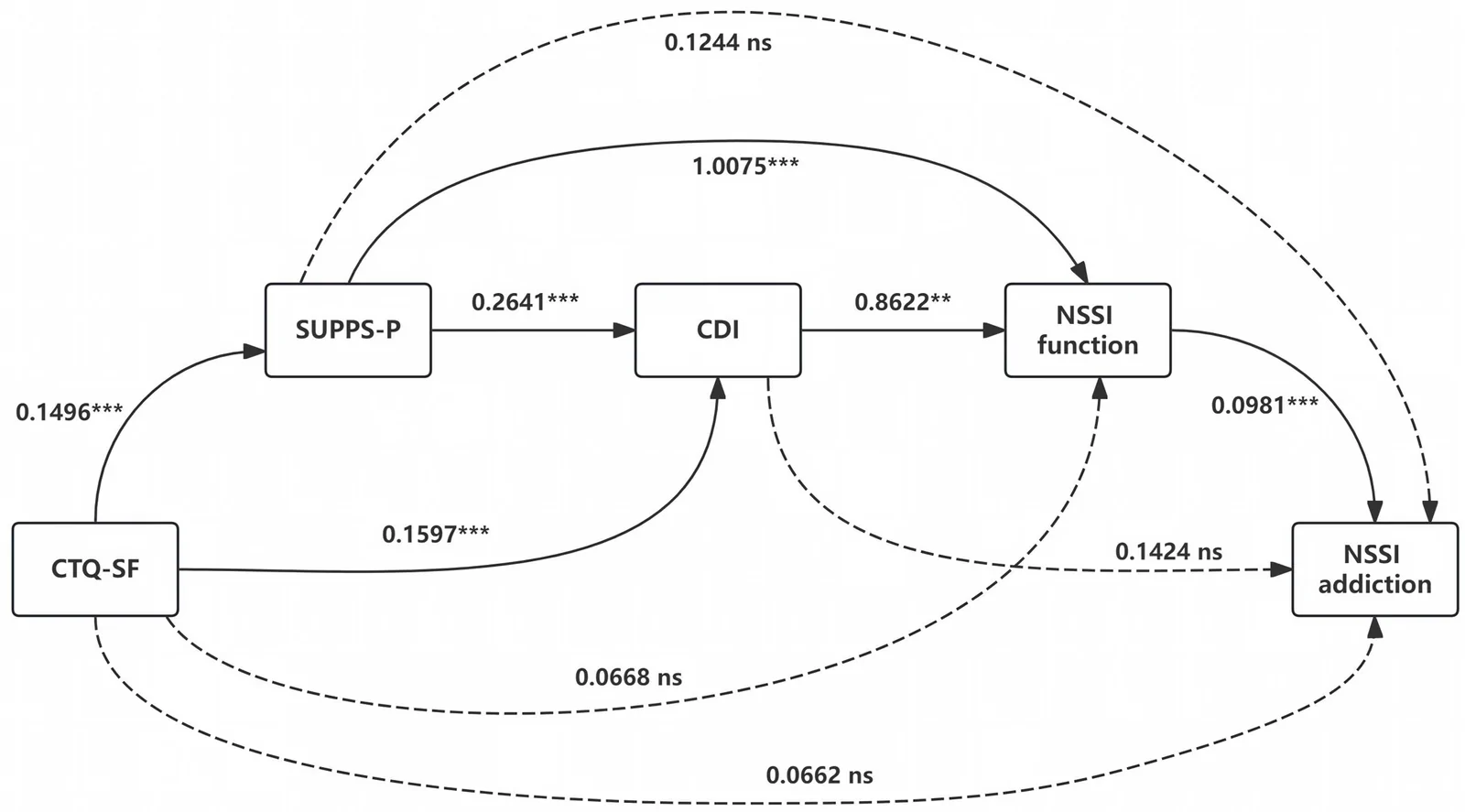
Path analysis model linking childhood trauma to NSSI addiction through impulsivity, depression, and NSSI function (*N* = 163). This figure displays all estimated paths in the serial mediation model examining the effect of childhood trauma (CTQ-SF) on NSSI addiction through impulsivity (SUPPS-P), depression (CDI), and NSSI function. Values shown are unstandardized coefficients (*B*) taken from the corresponding regression equations of the serial mediation model; each coefficient represents the effect of the predictor on that outcome, controlling for the other predictors of the outcome and for covariates. Solid lines indicate statistically significant paths (*p* < 0.05), whereas dashed lines indicate non-significant paths. Significance levels: *p < 0.05, **p < 0.01, ***p < 0.001; ns = not significant. The direct effect of CTQ-SF on NSSI addiction was marginally non-significant (*c*′ = 0.0662, *p* = 0.052), as were the paths from SUPPS-P (*p* = 0.055) and CDI (*p* = 0.056) to NSSI addiction, indicating that the association was predominantly, though not entirely, mediated. The total indirect effect was 0.0851 (95% CI [0.0366, 0.1538]), accounting for 56.2% of the total effect. The full sequential path (CTQ-SF → SUPPS-P → CDI → NSSI function → NSSI addiction) was significant (Ind7 = 0.0033, 95% CI [0.0007, 0.0083]), providing evidence for the hypothesized cascade from distal risk (childhood trauma) to proximal trait (impulsivity), then to emotional state (depression), followed by behavioral function (NSSI function), and ultimately to addictive behavior. Covariates (gender, age, years of education, illness duration, number of MDD episodes, and parental marital status) were included in the model but are not shown for simplicity. CTQ-SF, Childhood Trauma Questionnaire – Short Form; SUPPS-P, Short UPPS-P Impulsive Behavior Scale; CDI, Children’s Depression Inventory; NSSI, non-suicidal self-injury.

The full sequential path (CTQ-SF → SUPPS-P → CDI → OSI Functions → OSI Addictive Features) was significant (*Ind7* = 0.0033, 95% CI [0.0007, 0.0083]). Two-mediator chains including NSSI function were also significant: CTQ-SF → SUPPS-P → OSI Functions → addiction (*Ind5* = 0.0148, 95% CI [0.0039, 0.0336]) and CTQ-SF → CDI → OSI Functions → addiction (*Ind6* = 0.0135, 95% CI [0.0038, 0.0302]). However, none of the remaining indirect effects were significant: the chain without NSSI function (CTQ-SF → SUPPS-P → CDI → addiction; *Ind4* = 0.0056, 95% CI [-0.0006, 0.0169]) and the simple mediations via impulsivity alone (*Ind1* = 0.0186, 95% CI [-0.0002, 0.0477]), depression alone (*Ind2* = 0.0227, 95% CI [-0.0030, 0.0545]), and NSSI function alone (*Ind3* = 0.0066, 95% CI [-0.0169, 0.0345]). Thus, only indirect effects that passed through NSSI function in combination with at least one upstream mediator (*Ind5*–*Ind7*) were significant, indicating that no single mediator was sufficient to transmit the association between childhood trauma and NSSI addiction. This pattern was unchanged when parental marital status was removed from the covariate set (**Supplementary Materials**).

Collectively, these findings demonstrate that childhood trauma influences NSSI addiction through a sequential cascade, primarily via the pathway: childhood trauma → impulsivity → depression → NSSI function → NSSI addiction. The total indirect effect accounted for 56.2% of the total effect, supporting the hypothesized “distal risk → proximal trait → emotional state → behavioral coping → addictive outcome” mechanism.

### 3.5 Sensitivity and exploratory analyses

Given the multidimensionality of impulsivity, exploratory analyses replaced the SUPPS-P total score, in turn, with each of its five facets (negative urgency, positive urgency, sensation seeking, lack of perseverance, and lack of premeditation; **Supplementary Materials**). The four-item facet scores showed acceptable internal consistency for four facets (Cronbach’s *α* = 0.665–0.803) but low consistency for negative urgency (*α* = 0.549). Across all five models, the indirect effect through depression and NSSI function (CTQ-SF → CDI → OSI Functions → addiction) remained significant, whereas facet-involving indirect effects were significant only for positive urgency—which reproduced the full five-step cascade (*Ind7* = 0.0022, 95% CI [0.0001, 0.0064]) and the trauma → impulsivity → NSSI function chain (*Ind5* = 0.0094, 95% CI [0.0007, 0.0252])—and for sensation seeking, which showed a significant two-step path through NSSI function (*Ind5* = 0.0113, 95% CI [0.0021, 0.0263]). None of the indirect paths involving negative urgency, lack of perseverance, or lack of premeditation was significant.

When the order of the first two mediators was reversed (childhood trauma → depression → impulsivity → NSSI function → NSSI addiction), the total and direct effects were identical to those of the primary model, and the full sequential path remained significant (*Ind7* = 0.0072, 95% CI [0.0021, 0.0164]; **Supplementary Materials**). Thus, both orderings are compatible with the data; given the cross-sectional design, the preferred trait-before-state ordering rests on theoretical grounds (34, 35) rather than on empirical adjudication.

Finally, excluding parental marital status from the covariate set did not alter the core conclusions: the function-involving indirect effects (*Ind5*, *Ind6*) and the full sequential path (*Ind7*) remained significant in all three imputations, and the direct effect was non-significant throughout this reduced specification (**Supplementary Tables S8**-**S10**). The simple impulsivity-only path (*Ind1*) reached significance in two of the three imputations in this specification, but with bootstrap lower bounds close to zero and a similarly marginal lower bound in the primary model (−0.0002), so this difference should be interpreted with caution.

## 4 Discussion

The present study tested a trait-state sequential mediation model in 163 adolescents with MDD and NSSI. Because the present data are cross-sectional, the pathways reported here should be interpreted as a theoretically plausible sequence rather than as evidence of a causal chain; longitudinal designs are needed to establish temporal precedence. Results supported the hypothesized cascade: childhood trauma (distal risk) → trait impulsivity (proximal vulnerability) → depressive state → NSSI function (maladaptive coping) → NSSI addiction (addictive outcome). The full sequential path was significant, with the total indirect effect accounting for 56.2% of the total effect, and the direct effect being attenuated to marginal non-significance (*p* = 0.052), indicating that the association was predominantly, though not entirely, mediated. Unlike prior research examining isolated associations, our study provides a comprehensive, theory-driven understanding of how distal adversity translates into behavioral addiction.

The significant five-step path (*Ind7* = 0.0033, 95% CI [0.0007, 0.0083]) supports the premise that addiction arises from a multi-stage process: within this framework, an environmental risk factor may predispose toward a stable disposition, which in turn may precipitate a negative emotional state; this negative state is associated with a reinforcing behavioral strategy that can become compulsive (48, 49). Each individual link in this chain was statistically significant. Consistent with prior work, childhood trauma was positively associated with trait impulsivity (22, 23), and trait impulsivity predicted greater depressive severity (34, 35). Furthermore, higher depressive symptoms were associated with stronger endorsement of the reinforcement functions of NSSI (27), and this functional coping strategy was directly linked to greater addictive features (29). Thus, our results extend previous research by integrating these isolated links into a single sequential model and by demonstrating that the complete cascade is necessary to explain the trauma-addiction relationship.

Prior work has consistently implicated impulsivity and depression in the link between childhood trauma and NSSI. For example, a study of 160 hospitalized adolescents with NSSI found that those with a history of childhood maltreatment exhibited significantly higher trait impulsivity, which in turn was associated with more frequent self-injury and more severe clinical outcomes (24); a large community-based study of 3,918 young men reported that impulsivity partially mediated the effect of child maltreatment on NSSI, with indirect effects accounting for 34.3% of the total effect in individuals without suicidal self-injury and 51.2% in those with suicidal self-injury (50). Additionally, it has been demonstrated that depression also mediates the trauma-NSSI pathway, with mediation proportions of 45.5%–53.9% in a large Chinese adolescent sample (51) and independent associations in adolescents with mood disorders (52). These findings converge with the present results in identifying impulsivity and depression as key upstream vulnerabilities translating distal childhood adversity into proximal self-injurious outcomes; in our model, however, their contribution emerged only in combination with the reinforcing function of NSSI (*Ind5*–*Ind7*) rather than as simple mediations (*Ind1*–*Ind2*). Notably, no prior study has examined these mediators specifically in the context of NSSI addiction, extending the established mediation model to a more severe and compulsive form of self-injury.

None of the simple mediations—via impulsivity alone (*Ind1*), depression alone (*Ind2*), or NSSI function alone (*Ind3*)—was significant. These null findings suggest that no single mediator is sufficient: NSSI function does not operate in isolation, and the upstream trait and state vulnerabilities likewise require the downstream behavioral mechanism. According to the four-function model (53, 54), transforming a functional motivation into addiction depends on repeated reinforcement over time—which is unlikely without a pre-existing impulsive trait and a sustained depressive state (5). Critically, the impulsivity–depression pathway without NSSI function (*Ind4*) was also non-significant. This indicates that even the combination of trait impulsivity and depressive state—without the behavioral act of self-injury—is insufficient to mediate the trauma–addiction link. A key distinction from prior research is that most earlier studies treated impulsivity and depression as parallel or single mediators (50, 51), whereas we tested their sequential ordering. Our findings suggest that impulsivity acts as a predisposing trait that, when activated by a depressive state, requires the behavioral act of self-injury (and its reinforcing consequences) to translate into full-blown addiction.

Although the full five-step indirect effect (*Ind7* = 0.0033) is small in absolute magnitude, this reflects the multiplicative nature of serial mediation, in which each successive path coefficient compounds to attenuate the size of any single indirect path. The small magnitude of *Ind7* should therefore not be interpreted in isolation; rather, the meaningful signal lies in the consistent emergence of the complete cascade (*Ind5*, *Ind6*, and *Ind7*) alongside the null findings for the truncated chains (*Ind1*–*Ind4*), which together indicate that the behavioral act of NSSI—preceded by trait and state vulnerabilities—is a necessary link in the pathway from childhood trauma to addictive features. Clinically, even a small but reliable cascade may matter at the population level, and interventions targeting any single link (impulsivity, depression, or the reinforcing function of NSSI) could interrupt the progression toward addiction.

Among the mediators, the NSSI function played a critical but conditional role, emerging only when combined with preceding trait or state vulnerabilities. The two-step chains that included the NSSI function (*Ind5* and *Ind6*) were both significant, capturing the learning process by which functional reinforcement escalates into addiction. In contrast, the single-step functional pathway (*Ind3*) was inadequate. Consistent with the I-PACE model (48), affective responses to person-level characteristics drive addictive behaviors. NSSI function should therefore be conceptualized as a behavioral bridge translating trait-state vulnerabilities into compulsive self-injury, while depression and impulsivity operate as necessary upstream vulnerabilities.

The exploratory facet-level analyses add nuance to the role of impulsivity. When positive urgency replaced the SUPPS-P total score, the full five-step cascade remained significant, and sensation seeking retained a significant two-step path through NSSI function; in contrast, none of the indirect paths involving negative urgency, lack of perseverance, or lack of premeditation was significant. Although this pattern was not anticipated—negative urgency and lack of premeditation are often highlighted as the facets most relevant to NSSI (18, 19)—it should be interpreted with caution given the modest reliability of the four-item facet scores (Cronbach’s *α* = 0.549–0.803) and the exploratory nature of these analyses. Measurement error associated with brief facet scales may attenuate the corresponding indirect effects, and facet-level conclusions therefore await replication with longer instruments.

Our findings have several clinical implications. First, childhood trauma should be routinely assessed in adolescents with NSSI, as the present study suggests that early-life trauma may serve as a distal risk factor that, in theory, sets in motion a cascade toward impulsivity, depression, and eventually NSSI addiction. Second, within early intervention for NSSI and NSSI addiction, trait impulsivity should be specifically targeted as a proximal vulnerability factor. Third, given that depressive symptoms formed part of the significant sequential pathways (in combination with the reinforcing function of NSSI), effective treatment of these symptoms is essential for preventing NSSI addiction. Fourth, comprehensive functional assessment of NSSI should be standard practice for depressed adolescents who self-injure. Clinicians can use tools such as the OSI Functions subscale to identify specific reinforcement mechanisms. Once identified, interventions teaching alternative coping strategies serving the same function—such as mindfulness, distress tolerance, and interpersonal effectiveness—may weaken the reinforcement of self-injury and prevent the progression to compulsive, addictive self-injury.

Several limitations should be acknowledged. First, the cross-sectional design precludes causal inferences; longitudinal studies are needed to confirm directionality. Second, self-report data may be subject to recall bias, though Harman’s test indicated common method bias was not severe. Third, the sample comprised Chinese adolescents with MDD and NSSI recruited from a single clinical center, and was predominantly female, limiting generalizability to other cultures, non-clinical populations, or males. Fourth, the small indirect effect of the full chain (*Ind7* = 0.0033) should be interpreted cautiously, though it is statistically significant and theoretically meaningful; relatedly, the proportion of the total effect accounted for by the indirect pathways (56.2%) is a descriptive summary subject to sampling uncertainty and should be viewed as approximate rather than as a precise estimate. Future research should replicate these findings with larger samples, longitudinal designs, and objective measures.

## 5 Conclusion

The present study provides empirical support for a serial mediation model in which childhood trauma is associated with NSSI addiction through a theorized sequential cascade: distal childhood trauma → proximal trait impulsivity → depressive state → NSSI as a reinforcing coping strategy → addictive self-injury. The total indirect effect accounted for 56.2% of the total effect. The non-significant pathways (*Ind1*–*Ind4*) further underscore that NSSI function cannot act alone, nor can impulsivity and depression without the behavioral act of self-injury. These findings highlight the importance of targeting impulsivity, depression, and particularly the reinforcing functions of self-injury in the prevention and treatment of NSSI addiction. By elucidating the trait-state-behavior-addiction pathway, our study advances theoretical understanding of NSSI addiction and offers actionable clinical targets for adolescents with MDD and addictive features of NSSI. These findings should be interpreted in light of the sample characteristics: participants were Chinese adolescents with MDD and a history of NSSI recruited from a single clinical center, and individuals with comorbid psychiatric conditions were excluded. Consequently, the results may not generalize to other cultural contexts, diagnostic groups, or community samples; replication in larger, multi-site, and culturally diverse samples is warranted.

## Data Availability

The raw data supporting the conclusions of this article will be made available by the authors, without undue reservation.
