# Supplementary material for: Trait, state, and behavior in adolescent NSSI addiction: a serial mediation model linking childhood trauma to NSSI addiction
Source: Front Psychiatry. 2026 Sep 28;17:1942023. doi: 10.3389/fpsyt.2026.1942023 (PMC13655704; doi:10.3389/fpsyt.2026.1942023)
Supplement: Supplementary file 1 [file Table1.docx]

**Supplementary Materials**

**Trait, State, and Behavior in Adolescent NSSI Addiction: A Serial Mediation Model Linking Childhood Trauma to NSSI Addiction**

All supplementary analyses were conducted with SPSS 27.0 and the PROCESS macro v5.0 (5,000 bootstrap resamples). Unless stated otherwise, models controlled for gender, age, years of education, total illness duration (months), number of MDD episodes, and parental marital status (two dummy variables, married as reference), and were based on the multiple imputation dataset (m = 10, Fully Conditional Specification) that included parental marital status. Imputation_1 was used for primary analyses; Imputation_5 and Imputation_10 served as robustness checks.

**Supplementary Table S1. Normality tests for core variables (Imputation_1, *N* = 163)**

| **Variable** | **Kolmogorov–Smirnov Z** | ***p*** | **Shapiro–Wilk W** | ***p*** |
| --- | --- | --- | --- | --- |
| CTQ-SF total score | 0.049 | .200* | 0.983 | 0.038 |
| SUPPS-P total score | 0.078 | 0.016 | 0.991 | 0.398 |
| CDI total score | 0.050 | .200* | 0.991 | 0.386 |
| OSI Functions subscale total score | 0.055 | .200* | 0.980 | 0.016 |
| OSI Addictive Features subscale total score | 0.058 | .200* | 0.983 | 0.039 |

Note. CTQ-SF = Childhood Trauma Questionnaire – Short Form; SUPPS-P = Short UPPS-P Impulsive Behavior Scale; CDI = Children’s Depression Inventory; OSI = Ottawa Self-Injury Inventory. Kolmogorov–Smirnov tests used the Lilliefors significance correction; *p* values reported as “.200” are lower bounds, indicating *p* > 0.200. Minor deviations from normality motivated the use of Spearman rank-order correlations and bootstrap inference throughout.

**Supplementary Table S2. Demographic and clinical characteristics of the sample (Imputation_5, *N* = 163)**

| **Variable** | **Mean (*SD*) / *n* (%)** | **Median** | **Min** | **Max** | **Skewness** | **Kurtosis** |
| --- | --- | --- | --- | --- | --- | --- |
| Demographic characteristics |  |  |  |  |  |  |
| Age (years) | 14.80 (1.68) | 15.00 | 12 | 18 | 0.24 | -0.94 |
| Gender (male) | 35 (21.5%) | — | — | — | — | — |
| Years of education | 8.95 (1.70) | 9.00 | 6 | 13 | 0.26 | -0.95 |
| Parental marital status |  |  |  |  |  |  |
| — Married | 107 (65.6%) | — | — | — | — | — |
| — Divorced | 48 (29.4%) | — | — | — | — | — |
| — Other | 8 (4.9%) | — | — | — | — | — |
| Clinical characteristics |  |  |  |  |  |  |
| Total MDD duration (months) | 19.66 (15.04) | 12.00 | 1 | 72 | 0.83 | 0.14 |
| Number of MDD episodes | 1.12 (0.41) | 1.00 | 1 | 4 | 4.22 | 20.76 |
| NSSI frequency (past year) |  |  |  |  |  |  |
| — 1–5 times | 48 (29.4%) | — | — | — | — | — |
| — Monthly | 43 (26.4%) | — | — | — | — | — |
| — Weekly | 57 (35.0%) | — | — | — | — | — |
| — Daily | 15 (9.2%) | — | — | — | — | — |
| CTQ-SF total score | 54.28 (15.03) | 54.35 | 25 | 94 | 0.33 | -0.18 |
| SUPPS-P total score | 51.17 (7.91) | 51.00 | 24.21 | 71 | -0.19 | 0.48 |
| CDI total score | 33.68 (7.25) | 34.00 | 14.54 | 54 | -0.15 | 0.18 |
| OSI Functions subscale total score | 46.61 (23.34) | 45.24 | 0 | 128 | 0.51 | 0.21 |
| OSI Addictive Features subscale total score | 13.48 (6.80) | 13.00 | 0 | 28 | 0.10 | -0.69 |

Note. *SD* = standard deviation; MDD = major depressive disorder; NSSI = non-suicidal self-injury. All values are based on Imputation_5 of the multiple imputation (m = 10) that included parental marital status. This table reproduces the structure of Table 1 in the main text for robustness-check purposes.

**Supplementary Table S3. Demographic and clinical characteristics of the sample (Imputation_10, *N* = 163)**

| **Variable** | **Mean (*SD*) / *n* (%)** | **Median** | **Min** | **Max** | **Skewness** | **Kurtosis** |
| --- | --- | --- | --- | --- | --- | --- |
| Demographic characteristics |  |  |  |  |  |  |
| Age (years) | 14.80 (1.68) | 15.00 | 12 | 18 | 0.24 | -0.94 |
| Gender (male) | 35 (21.5%) | — | — | — | — | — |
| Years of education | 8.95 (1.70) | 9.00 | 6 | 13 | 0.26 | -0.95 |
| Parental marital status |  |  |  |  |  |  |
| — Married | 106 (65.0%) | — | — | — | — | — |
| — Divorced | 49 (30.1%) | — | — | — | — | — |
| — Other | 8 (4.9%) | — | — | — | — | — |
| Clinical characteristics |  |  |  |  |  |  |
| Total MDD duration (months) | 19.72 (15.03) | 12.00 | 1 | 72 | 0.82 | 0.13 |
| Number of MDD episodes | 1.12 (0.41) | 1.00 | 1 | 4 | 4.22 | 20.76 |
| NSSI frequency (past year) |  |  |  |  |  |  |
| — 1–5 times | 50 (30.7%) | — | — | — | — | — |
| — Monthly | 42 (25.8%) | — | — | — | — | — |
| — Weekly | 57 (35.0%) | — | — | — | — | — |
| — Daily | 14 (8.6%) | — | — | — | — | — |
| CTQ-SF total score | 54.06 (15.09) | 54.00 | 25 | 94 | 0.34 | -0.18 |
| SUPPS-P total score | 51.18 (7.87) | 51.00 | 24.21 | 71 | -0.18 | 0.53 |
| CDI total score | 33.65 (7.24) | 34.00 | 14.54 | 54 | -0.14 | 0.20 |
| OSI Functions subscale total score | 47.10 (22.94) | 46.93 | 0 | 128 | 0.50 | 0.32 |
| OSI Addictive Features subscale total score | 13.48 (6.80) | 13.00 | 0 | 28 | 0.10 | -0.68 |

Note. *SD* = standard deviation; MDD = major depressive disorder; NSSI = non-suicidal self-injury. All values are based on Imputation_10 of the multiple imputation (m = 10) that included parental marital status. This table reproduces the structure of Table 1 in the main text for robustness-check purposes.

**Supplementary Table S4. Spearman’s rank-order correlation matrix among core variables (Imputation_5, *N* = 163)**

| **Variable** | **1** | **2** | **3** | **4** | **5** |
| --- | --- | --- | --- | --- | --- |
| 1. CTQ-SF | — |  |  |  |  |
| 2. SUPPS-P | 0.249** [0.094, 0.392] | — |  |  |  |
| 3. CDI | 0.392** [0.249, 0.518] | 0.388** [0.245, 0.514] | — |  |  |
| 4. OSI Functions subscale | 0.205** [0.048, 0.352] | 0.388** [0.245, 0.514] | 0.424** [0.285, 0.546] | — |  |
| 5. OSI Addictive Features | 0.333** [0.185, 0.467] | 0.391** [0.248, 0.517] | 0.432** [0.294, 0.552] | 0.489** [0.359, 0.601] | — |

Note. Values are Spearman’s rank-order correlation coefficients with 95% confidence intervals in brackets based on Fisher’s z transformation with the Fieller–Hartley–Pearson standard-error approximation for rank correlations. All values are based on Imputation_5. **p* < 0.05, ***p* < 0.01 (two-tailed).

**Supplementary Table S5. Spearman’s rank-order correlation matrix among core variables (Imputation_10, *N* = 163)**

| **Variable** | **1** | **2** | **3** | **4** | **5** |
| --- | --- | --- | --- | --- | --- |
| 1. CTQ-SF | — |  |  |  |  |
| 2. SUPPS-P | 0.232** [0.076, 0.376] | — |  |  |  |
| 3. CDI | 0.371** [0.227, 0.500] | 0.367** [0.222, 0.496] | — |  |  |
| 4. OSI Functions subscale | 0.183* [0.025, 0.331] | 0.347** [0.200, 0.479] | 0.416** [0.276, 0.539] | — |  |
| 5. OSI Addictive Features | 0.342** [0.194, 0.474] | 0.372** [0.228, 0.501] | 0.434** [0.296, 0.554] | 0.476** [0.344, 0.590] | — |

Note. Values are Spearman’s rank-order correlation coefficients with 95% confidence intervals in brackets based on Fisher’s z transformation with the Fieller–Hartley–Pearson standard-error approximation for rank correlations. All values are based on Imputation_10. **p* < 0.05, ***p* < 0.01 (two-tailed).

**Supplementary Table S6. Serial mediation analysis of the pathway from childhood trauma to NSSI addiction (Imputation_5; 5,000 bootstrap resamples)**

| **Path** | **Effect (*B*)** | **Boot *SE*** | **95% CI** | ***p*** | **Proportion mediated** |
| --- | --- | --- | --- | --- | --- |
| Total effect (*c*) | 0.1473 | 0.0353 | [0.0775, 0.2170] | <0.001 | 100% |
| Direct effect (*c*′) | 0.0640 | 0.0334 | [-0.0019, 0.1299] | 0.057 | — |
| Total indirect | 0.0832 | 0.0300 | [0.0329, 0.1512] | — | 56.5% * |
| Ind1: CTQ-SF → SUPPS-P → Addictive Features | 0.0148 | 0.0108 | [-0.0016, 0.0404] | — | — |
| Ind2: CTQ-SF → CDI → Addictive Features | 0.0223 | 0.0147 | [-0.0048, 0.0538] | — | — |
| Ind3: CTQ-SF → OSI Functions → Addictive Features | 0.0091 | 0.0151 | [-0.0184, 0.0428] | — | — |
| Ind4: CTQ-SF → SUPPS-P → CDI → Addictive Features | 0.0050 | 0.0042 | [-0.0009, 0.0152] | — | — |
| Ind5: CTQ-SF → SUPPS-P → OSI Functions → Addictive Features | 0.0143 | 0.0075 | [0.0038, 0.0323] | — | 9.7% * |
| Ind6: CTQ-SF → CDI → OSI Functions → Addictive Features | 0.0145 | 0.0072 | [0.0043, 0.0320] | — | 9.8% * |
| Ind7: CTQ-SF → SUPPS-P → CDI → OSI Functions → Addictive Features | 0.0033 | 0.0019 | [0.0007, 0.0080] | — | 2.2% * |

Note. *N* = 163. Covariates: gender, age, years of education, total illness duration (months), number of MDD episodes, and parental marital status (two dummy variables, married as reference). 95% CIs for indirect effects are percentile bootstrap CIs based on 5,000 resamples; *p* values for the total and direct effects are from ordinary least squares (OLS) *t* tests. Proportion mediated = indirect effect / total effect; shown only for significant indirect effects (marked *), i.e., 95% bootstrap CI excluding zero. This table is the robustness-check counterpart of Table 4 in the main text, which reports Imputation_1.

**Supplementary Table S7. Serial mediation analysis of the pathway from childhood trauma to NSSI addiction (Imputation_10; 5,000 bootstrap resamples)**

| **Path** | **Effect (*B*)** | **Boot *SE*** | **95% CI** | ***p*** | **Proportion mediated** |
| --- | --- | --- | --- | --- | --- |
| Total effect (*c*) | 0.1460 | 0.0352 | [0.0765, 0.2155] | <0.001 | 100% |
| Direct effect (*c*′) | 0.0716 | 0.0330 | [0.0065, 0.1368] | 0.031 | — |
| Total indirect | 0.0744 | 0.0288 | [0.0246, 0.1375] | — | 51.0% * |
| Ind1: CTQ-SF → SUPPS-P → Addictive Features | 0.0148 | 0.0106 | [-0.0002, 0.0404] | — | — |
| Ind2: CTQ-SF → CDI → Addictive Features | 0.0221 | 0.0139 | [-0.0038, 0.0502] | — | — |
| Ind3: CTQ-SF → OSI Functions → Addictive Features | 0.0035 | 0.0145 | [-0.0237, 0.0350] | — | — |
| Ind4: CTQ-SF → SUPPS-P → CDI → Addictive Features | 0.0049 | 0.0040 | [-0.0007, 0.0147] | — | — |
| Ind5: CTQ-SF → SUPPS-P → OSI Functions → Addictive Features | 0.0118 | 0.0070 | [0.0025, 0.0290] | — | 8.1% * |
| Ind6: CTQ-SF → CDI → OSI Functions → Addictive Features | 0.0142 | 0.0071 | [0.0041, 0.0315] | — | 9.7% * |
| Ind7: CTQ-SF → SUPPS-P → CDI → OSI Functions → Addictive Features | 0.0031 | 0.0019 | [0.0006, 0.0079] | — | 2.1% * |

Note. *N* = 163. Covariates: gender, age, years of education, total illness duration (months), number of MDD episodes, and parental marital status (two dummy variables, married as reference). 95% CIs for indirect effects are percentile bootstrap CIs based on 5,000 resamples; *p* values for the total and direct effects are from OLS *t*-tests. Proportion mediated = indirect effect / total effect; shown only for significant indirect effects (marked *), i.e., 95% bootstrap CI excluding zero. This table is the robustness-check counterpart of Table 4 in the main text, which reports Imputation_1.

**Supplementary Table S8. Sensitivity analysis: serial mediation excluding parental marital status from the covariate set (Imputation_1; 5,000 bootstrap resamples)**

| **Path** | **Effect (*B*)** | **Boot *SE*** | **95% CI** | ***p*** | **Proportion mediated** |
| --- | --- | --- | --- | --- | --- |
| Total effect (*c*) | 0.1366 | 0.0344 | [0.0686, 0.2045] | <0.001 | 100% |
| Direct effect (*c*′) | 0.0530 | 0.0326 | [-0.0114, 0.1174] | 0.106 | — |
| Total indirect | 0.0836 | 0.0272 | [0.0379, 0.1444] | — | 61.2% * |
| Ind1: CTQ-SF → SUPPS-P → Addictive Features | 0.0180 | 0.0113 | [0.0008, 0.0445] | — | 13.2% * |
| Ind2: CTQ-SF → CDI → Addictive Features | 0.0228 | 0.0134 | [-0.0014, 0.0517] | — | — |
| Ind3: CTQ-SF → OSI Functions → Addictive Features | 0.0080 | 0.0124 | [-0.0156, 0.0344] | — | — |
| Ind4: CTQ-SF → SUPPS-P → CDI → Addictive Features | 0.0057 | 0.0043 | [-0.0003, 0.0163] | — | — |
| Ind5: CTQ-SF → SUPPS-P → OSI Functions → Addictive Features | 0.0135 | 0.0075 | [0.0035, 0.0323] | — | 9.9% * |
| Ind6: CTQ-SF → CDI → OSI Functions → Addictive Features | 0.0123 | 0.0064 | [0.0033, 0.0279] | — | 9.0% * |
| Ind7: CTQ-SF → SUPPS-P → CDI → OSI Functions → Addictive Features | 0.0031 | 0.0019 | [0.0007, 0.0079] | — | 2.3% * |

Note. *N* = 163. Covariates: gender, age, years of education, total illness duration (months), and number of MDD episodes; parental marital status was omitted from the covariate set in order to isolate its influence on the estimates. 95% CIs for indirect effects are percentile bootstrap CIs based on 5,000 resamples; *p* values for the total and direct effects are from OLS *t* tests. Proportion mediated = indirect effect / total effect; shown only for significant indirect effects (marked *), i.e., 95% bootstrap CI excluding zero. This sensitivity analysis examines whether the inclusion of parental marital status as a covariate materially altered the conclusions. The function-involving indirect effects (Ind5, Ind6) and the full sequential path (Ind7) were significant in all three imputations, as in the primary model. The simple impulsivity-only path (Ind1) reached significance in two of the three imputations in this reduced specification, albeit with bootstrap lower bounds close to zero (in the primary model its lower bound was similarly marginal, −0.0002), and the direct effect was non-significant throughout this specification.

**Supplementary Table S9. Sensitivity analysis: serial mediation excluding parental marital status from the covariate set (Imputation_5; 5,000 bootstrap resamples)**

| **Path** | **Effect (*B*)** | **Boot *SE*** | **95% CI** | ***p*** | **Proportion mediated** |
| --- | --- | --- | --- | --- | --- |
| Total effect (*c*) | 0.1336 | 0.0343 | [0.0658, 0.2013] | <0.001 | 100% |
| Direct effect (*c*′) | 0.0549 | 0.0320 | [-0.0084, 0.1182] | 0.089 | — |
| Total indirect | 0.0787 | 0.0271 | [0.0322, 0.1384] | — | 58.9% * |
| Ind1: CTQ-SF → SUPPS-P → Addictive Features | 0.0138 | 0.0097 | [-0.0009, 0.0364] | — | — |
| Ind2: CTQ-SF → CDI → Addictive Features | 0.0219 | 0.0137 | [-0.0030, 0.0515] | — | — |
| Ind3: CTQ-SF → OSI Functions → Addictive Features | 0.0081 | 0.0138 | [-0.0186, 0.0377] | — | — |
| Ind4: CTQ-SF → SUPPS-P → CDI → Addictive Features | 0.0049 | 0.0041 | [-0.0006, 0.0152] | — | — |
| Ind5: CTQ-SF → SUPPS-P → OSI Functions → Addictive Features | 0.0132 | 0.0072 | [0.0034, 0.0314] | — | 9.9% * |
| Ind6: CTQ-SF → CDI → OSI Functions → Addictive Features | 0.0138 | 0.0068 | [0.0040, 0.0307] | — | 10.3% * |
| Ind7: CTQ-SF → SUPPS-P → CDI → OSI Functions → Addictive Features | 0.0031 | 0.0018 | [0.0007, 0.0078] | — | 2.3% * |

Note. *N* = 163. Covariates: gender, age, years of education, total illness duration (months), and number of MDD episodes; parental marital status was omitted from the covariate set in order to isolate its influence on the estimates. 95% CIs for indirect effects are percentile bootstrap CIs based on 5,000 resamples; *p* values for the total and direct effects are from OLS *t*-tests. Proportion mediated = indirect effect / total effect; shown only for significant indirect effects (marked *), i.e., 95% bootstrap CI excluding zero. This sensitivity analysis examines whether the inclusion of parental marital status as a covariate materially altered the conclusions. The function-involving indirect effects (Ind5, Ind6) and the full sequential path (Ind7) were significant in all three imputations, as in the primary model. The simple impulsivity-only path (Ind1) reached significance in two of the three imputations in this reduced specification, albeit with bootstrap lower bounds close to zero (in the primary model, its lower bound was similarly marginal, −0.0002), and the direct effect was non-significant throughout this specification.

**Supplementary Table S10. Sensitivity analysis: serial mediation excluding parental marital status from the covariate set (Imputation_10; 5,000 bootstrap resamples)**

| **Path** | **Effect (*B*)** | **Boot *SE*** | **95% CI** | ***p*** | **Proportion mediated** |
| --- | --- | --- | --- | --- | --- |
| Total effect (*c*) | 0.1315 | 0.0342 | [0.0640, 0.1990] | <0.001 | 100% |
| Direct effect (*c*′) | 0.0599 | 0.0318 | [-0.0029, 0.1226] | 0.061 | — |
| Total indirect | 0.0716 | 0.0268 | [0.0251, 0.1303] | — | 54.4% * |
| Ind1: CTQ-SF → SUPPS-P → Addictive Features | 0.0138 | 0.0094 | [0.0002, 0.0361] | — | 10.5% * |
| Ind2: CTQ-SF → CDI → Addictive Features | 0.0221 | 0.0131 | [-0.0018, 0.0501] | — | — |
| Ind3: CTQ-SF → OSI Functions → Addictive Features | 0.0045 | 0.0143 | [-0.0207, 0.0358] | — | — |
| Ind4: CTQ-SF → SUPPS-P → CDI → Addictive Features | 0.0046 | 0.0039 | [-0.0003, 0.0143] | — | — |
| Ind5: CTQ-SF → SUPPS-P → OSI Functions → Addictive Features | 0.0104 | 0.0064 | [0.0020, 0.0271] | — | 7.9% * |
| Ind6: CTQ-SF → CDI → OSI Functions → Addictive Features | 0.0134 | 0.0065 | [0.0038, 0.0288] | — | 10.2% * |
| Ind7: CTQ-SF → SUPPS-P → CDI → OSI Functions → Addictive Features | 0.0028 | 0.0017 | [0.0005, 0.0070] | — | 2.1% * |

Note. *N* = 163. Covariates: gender, age, years of education, total illness duration (months), and number of MDD episodes; parental marital status was omitted from the covariate set in order to isolate its influence on the estimates. 95% CIs for indirect effects are percentile bootstrap CIs based on 5,000 resamples; *p* values for the total and direct effects are from OLS *t*-tests. Proportion mediated = indirect effect / total effect; shown only for significant indirect effects (marked *), i.e., 95% bootstrap CI excluding zero. This sensitivity analysis examines whether the inclusion of parental marital status as a covariate materially altered the conclusions. The function-involving indirect effects (Ind5, Ind6) and the full sequential path (Ind7) were significant in all three imputations, as in the primary model. The simple impulsivity-only path (Ind1) reached significance in two of the three imputations in this reduced specification, albeit with bootstrap lower bounds close to zero (in the primary model, its lower bound was similarly marginal, −0.0002), and the direct effect was non-significant throughout this specification.

**Supplementary Table S11. Descriptive statistics, internal consistency, and correlations with the SUPPS-P total score for the five impulsivity facets (Imputation_1, *N* = 163)**

| **Facet (four items each)** | ***M*** | ***SD*** | **Median** | **Min** | **Max** | **Cronbach’s α** | ***r* with total** |
| --- | --- | --- | --- | --- | --- | --- | --- |
| Negative urgency | 10.84 | 2.48 | 11.00 | 4 | 16 | 0.549 | 0.633 |
| Positive urgency | 10.64 | 2.83 | 11.00 | 4 | 16 | 0.757 | 0.765 |
| Sensation seeking | 9.03 | 2.98 | 9.00 | 4 | 16 | 0.722 | 0.591 |
| Lack of premeditation | 10.44 | 2.74 | 10.00 | 4 | 16 | 0.803 | 0.554 |
| Lack of perseverance | 10.25 | 2.24 | 10.00 | 4 | 16 | 0.665 | 0.389 |

Note. Facet scores were computed from the 20 SUPPS-P items after reverse-scoring; the total score showed Cronbach’s α = 0.769. Facet–total correlations are Spearman rank-order coefficients (all *p* < 0.001). Given only four items per facet, the internal consistencies of the facet scores are, as expected, lower than that of the 20-item total score; negative urgency showed relatively low consistency (α = 0.549), which should be considered when interpreting the facet-level analyses.

**Supplementary Table S12. Exploratory facet-level serial mediation analyses: each SUPPS-P facet replacing the total score as the first mediator (Imputation_1; 5,000 bootstrap resamples)**

| **Facet / path** | **Effect (*B*)** | **Boot *SE*** | **95% CI** | ***p*** | **Proportion mediated** |
| --- | --- | --- | --- | --- | --- |
| Negative urgency |  |  |  |  |  |
| Total effect (*c*) | 0.1428 | 0.0352 | [0.0733, 0.2123] | <0.001 | 100% |
| Direct effect (*c*′) | 0.0721 | 0.0323 | [0.0083, 0.1359] | 0.027 | — |
| Total indirect | 0.0707 | 0.0291 | [0.0221, 0.1346] | — | 49.5% |
| Ind1: CTQ-SF → Negative urgency → Addictive Features | 0.0030 | 0.0059 | [-0.0057, 0.0184] | — | — |
| Ind2: CTQ-SF → CDI → Addictive Features | 0.0298 | 0.0166 | [-0.0008, 0.0644] | — | — |
| Ind3: CTQ-SF → OSI Functions → Addictive Features | 0.0144 | 0.0162 | [-0.0134, 0.0511] | — | — |
| Ind4: CTQ-SF → Negative urgency → CDI → Addictive Features | 0.0006 | 0.0011 | [-0.0011, 0.0036] | — | — |
| Ind5: CTQ-SF → Negative urgency → OSI Functions → Addictive Features | 0.0027 | 0.0050 | [-0.0043, 0.0155] | — | — |
| Ind6: CTQ-SF → CDI → OSI Functions → Addictive Features | 0.0198 | 0.0083 | [0.0073, 0.0397] | — | 13.9% * |
| Ind7: CTQ-SF → Negative urgency → CDI → OSI Functions → Addictive Features | 0.0004 | 0.0008 | [-0.0007, 0.0023] | — | — |
| Positive urgency |  |  |  |  |  |
| Total effect (*c*) | 0.1428 | 0.0352 | [0.0733, 0.2123] | <0.001 | 100% |
| Direct effect (*c*′) | 0.0691 | 0.0324 | [0.0050, 0.1331] | 0.035 | — |
| Total indirect | 0.0737 | 0.0297 | [0.0223, 0.1395] | — | 51.6% |
| Ind1: CTQ-SF → Positive urgency → Addictive Features | 0.0070 | 0.0074 | [-0.0044, 0.0246] | — | — |
| Ind2: CTQ-SF → CDI → Addictive Features | 0.0245 | 0.0143 | [-0.0025, 0.0544] | — | — |
| Ind3: CTQ-SF → OSI Functions → Addictive Features | 0.0118 | 0.0161 | [-0.0169, 0.0490] | — | — |
| Ind4: CTQ-SF → Positive urgency → CDI → Addictive Features | 0.0035 | 0.0034 | [-0.0003, 0.0127] | — | — |
| Ind5: CTQ-SF → Positive urgency → OSI Functions → Addictive Features | 0.0094 | 0.0063 | [0.0007, 0.0252] | — | 6.6% * |
| Ind6: CTQ-SF → CDI → OSI Functions → Addictive Features | 0.0152 | 0.0075 | [0.0043, 0.0329] | — | 10.6% * |
| Ind7: CTQ-SF → Positive urgency → CDI → OSI Functions → Addictive Features | 0.0022 | 0.0016 | [0.0001, 0.0064] | — | 1.5% * |
| Sensation seeking |  |  |  |  |  |
| Total effect (*c*) | 0.1428 | 0.0352 | [0.0733, 0.2123] | <0.001 | 100% |
| Direct effect (*c*′) | 0.0650 | 0.0330 | [-0.0003, 0.1302] | 0.051 | — |
| Total indirect | 0.0778 | 0.0296 | [0.0284, 0.1445] | — | 54.5% |
| Ind1: CTQ-SF → Sensation seeking → Addictive Features | 0.0065 | 0.0080 | [-0.0070, 0.0246] | — | — |
| Ind2: CTQ-SF → CDI → Addictive Features | 0.0295 | 0.0165 | [0.0013, 0.0660] | — | 20.7% * |
| Ind3: CTQ-SF → OSI Functions → Addictive Features | 0.0052 | 0.0168 | [-0.0242, 0.0425] | — | — |
| Ind4: CTQ-SF → Sensation seeking → CDI → Addictive Features | 0.0019 | 0.0022 | [-0.0013, 0.0073] | — | — |
| Ind5: CTQ-SF → Sensation seeking → OSI Functions → Addictive Features | 0.0113 | 0.0064 | [0.0021, 0.0263] | — | 7.9% * |
| Ind6: CTQ-SF → CDI → OSI Functions → Addictive Features | 0.0219 | 0.0088 | [0.0081, 0.0421] | — | 15.3% * |
| Ind7: CTQ-SF → Sensation seeking → CDI → OSI Functions → Addictive Features | 0.0014 | 0.0015 | [-0.0009, 0.0052] | — | — |
| Lack of premeditation |  |  |  |  |  |
| Total effect (*c*) | 0.1428 | 0.0352 | [0.0733, 0.2123] | <0.001 | 100% |
| Direct effect (*c*′) | 0.0681 | 0.0326 | [0.0037, 0.1324] | 0.038 | — |
| Total indirect | 0.0747 | 0.0286 | [0.0258, 0.1391] | — | 52.3% |
| Ind1: CTQ-SF → Lack of premeditation → Addictive Features | 0.0037 | 0.0061 | [-0.0051, 0.0191] | — | — |
| Ind2: CTQ-SF → CDI → Addictive Features | 0.0279 | 0.0159 | [-0.0009, 0.0619] | — | — |
| Ind3: CTQ-SF → OSI Functions → Addictive Features | 0.0152 | 0.0190 | [-0.0171, 0.0592] | — | — |
| Ind4: CTQ-SF → Lack of premeditation → CDI → Addictive Features | 0.0018 | 0.0020 | [-0.0004, 0.0071] | — | — |
| Ind5: CTQ-SF → Lack of premeditation → OSI Functions → Addictive Features | -0.0001 | 0.0027 | [-0.0068, 0.0044] | — | — |
| Ind6: CTQ-SF → CDI → OSI Functions → Addictive Features | 0.0246 | 0.0097 | [0.0101, 0.0471] | — | 17.2% * |
| Ind7: CTQ-SF → Lack of premeditation → CDI → OSI Functions → Addictive Features | 0.0016 | 0.0013 | [-0.0004, 0.0047] | — | — |
| Lack of perseverance |  |  |  |  |  |
| Total effect (*c*) | 0.1428 | 0.0352 | [0.0733, 0.2123] | <0.001 | 100% |
| Direct effect (*c*′) | 0.0702 | 0.0326 | [0.0058, 0.1346] | 0.033 | — |
| Total indirect | 0.0725 | 0.0284 | [0.0233, 0.1357] | — | 50.8% |
| Ind1: CTQ-SF → Lack of perseverance → Addictive Features | -0.0014 | 0.0042 | [-0.0112, 0.0069] | — | — |
| Ind2: CTQ-SF → CDI → Addictive Features | 0.0312 | 0.0167 | [0.0013, 0.0674] | — | 21.8% * |
| Ind3: CTQ-SF → OSI Functions → Addictive Features | 0.0148 | 0.0189 | [-0.0173, 0.0583] | — | — |
| Ind4: CTQ-SF → Lack of perseverance → CDI → Addictive Features | 0.0013 | 0.0018 | [-0.0006, 0.0061] | — | — |
| Ind5: CTQ-SF → Lack of perseverance → OSI Functions → Addictive Features | 0.0010 | 0.0022 | [-0.0028, 0.0063] | — | — |
| Ind6: CTQ-SF → CDI → OSI Functions → Addictive Features | 0.0246 | 0.0096 | [0.0098, 0.0467] | — | 17.2% * |
| Ind7: CTQ-SF → Lack of perseverance → CDI → OSI Functions → Addictive Features | 0.0010 | 0.0011 | [-0.0005, 0.0040] | — | — |

Note. *N* = 163. Covariates: gender, age, years of education, total illness duration (months), number of MDD episodes, and parental marital status (two dummy variables, married as reference). In each model, the designated facet replaced the SUPPS-P total score as the first mediator (CTQ-SF → facet → CDI → OSI Functions → addiction); depression (CDI) and NSSI function (OSI Functions) remained the second and third mediators. 95% CIs for indirect effects are percentile bootstrap CIs based on 5,000 resamples; *p* values for the total and direct effects are from OLS *t*-tests. Proportion mediated = indirect effect / total effect; shown only for significant indirect effects (marked *), i.e., 95% bootstrap CI excluding zero.

**Supplementary Table S13. Exploratory facet-level serial mediation analyses: each SUPPS-P facet replacing the total score as the first mediator (Imputation_5; 5,000 bootstrap resamples)**

| **Facet / path** | **Effect (*B*)** | **Boot *SE*** | **95% CI** | ***p*** | **Proportion mediated** |
| --- | --- | --- | --- | --- | --- |
| Negative urgency |  |  |  |  |  |
| Total effect (*c*) | 0.1543 | 0.0354 | [0.0844, 0.2242] | <0.001 | 100% |
| Direct effect (*c*′) | 0.0804 | 0.0332 | [0.0149, 0.1459] | 0.017 | — |
| Total indirect | 0.0739 | 0.0291 | [0.0250, 0.1401] | — | 47.9% |
| Ind1: CTQ-SF → Negative urgency → Addictive Features | 0.0024 | 0.0054 | [-0.0059, 0.0167] | — | — |
| Ind2: CTQ-SF → CDI → Addictive Features | 0.0286 | 0.0178 | [-0.0032, 0.0674] | — | — |
| Ind3: CTQ-SF → OSI Functions → Addictive Features | 0.0196 | 0.0166 | [-0.0078, 0.0572] | — | — |
| Ind4: CTQ-SF → Negative urgency → CDI → Addictive Features | 0.0005 | 0.0011 | [-0.0011, 0.0032] | — | — |
| Ind5: CTQ-SF → Negative urgency → OSI Functions → Addictive Features | 0.0023 | 0.0048 | [-0.0051, 0.0138] | — | — |
| Ind6: CTQ-SF → CDI → OSI Functions → Addictive Features | 0.0201 | 0.0084 | [0.0071, 0.0392] | — | 13.0% * |
| Ind7: CTQ-SF → Negative urgency → CDI → OSI Functions → Addictive Features | 0.0003 | 0.0007 | [-0.0009, 0.0021] | — | — |
| Positive urgency |  |  |  |  |  |
| Total effect (*c*) | 0.1543 | 0.0354 | [0.0844, 0.2242] | <0.001 | 100% |
| Direct effect (*c*′) | 0.0754 | 0.0332 | [0.0098, 0.1411] | 0.025 | — |
| Total indirect | 0.0789 | 0.0293 | [0.0281, 0.1423] | — | 51.1% |
| Ind1: CTQ-SF → Positive urgency → Addictive Features | 0.0091 | 0.0086 | [-0.0048, 0.0297] | — | — |
| Ind2: CTQ-SF → CDI → Addictive Features | 0.0228 | 0.0147 | [-0.0048, 0.0529] | — | — |
| Ind3: CTQ-SF → OSI Functions → Addictive Features | 0.0149 | 0.0158 | [-0.0119, 0.0510] | — | — |
| Ind4: CTQ-SF → Positive urgency → CDI → Addictive Features | 0.0037 | 0.0035 | [-0.0007, 0.0132] | — | — |
| Ind5: CTQ-SF → Positive urgency → OSI Functions → Addictive Features | 0.0111 | 0.0067 | [0.0014, 0.0269] | — | 7.2% * |
| Ind6: CTQ-SF → CDI → OSI Functions → Addictive Features | 0.0149 | 0.0074 | [0.0043, 0.0329] | — | 9.7% * |
| Ind7: CTQ-SF → Positive urgency → CDI → OSI Functions → Addictive Features | 0.0024 | 0.0017 | [0.0002, 0.0065] | — | 1.6% * |
| Sensation seeking |  |  |  |  |  |
| Total effect (*c*) | 0.1543 | 0.0354 | [0.0844, 0.2242] | <0.001 | 100% |
| Direct effect (*c*′) | 0.0729 | 0.0339 | [0.0059, 0.1399] | 0.033 | — |
| Total indirect | 0.0814 | 0.0290 | [0.0309, 0.1443] | — | 52.8% |
| Ind1: CTQ-SF → Sensation seeking → Addictive Features | 0.0052 | 0.0084 | [-0.0096, 0.0239] | — | — |
| Ind2: CTQ-SF → CDI → Addictive Features | 0.0290 | 0.0175 | [-0.0018, 0.0668] | — | — |
| Ind3: CTQ-SF → OSI Functions → Addictive Features | 0.0090 | 0.0169 | [-0.0200, 0.0459] | — | — |
| Ind4: CTQ-SF → Sensation seeking → CDI → Addictive Features | 0.0017 | 0.0021 | [-0.0018, 0.0069] | — | — |
| Ind5: CTQ-SF → Sensation seeking → OSI Functions → Addictive Features | 0.0122 | 0.0067 | [0.0025, 0.0285] | — | 7.9% * |
| Ind6: CTQ-SF → CDI → OSI Functions → Addictive Features | 0.0230 | 0.0088 | [0.0087, 0.0435] | — | 14.9% * |
| Ind7: CTQ-SF → Sensation seeking → CDI → OSI Functions → Addictive Features | 0.0013 | 0.0015 | [-0.0012, 0.0050] | — | — |
| Lack of premeditation |  |  |  |  |  |
| Total effect (*c*) | 0.1543 | 0.0354 | [0.0844, 0.2242] | <0.001 | 100% |
| Direct effect (*c*′) | 0.0748 | 0.0333 | [0.0089, 0.1407] | 0.026 | — |
| Total indirect | 0.0795 | 0.0290 | [0.0307, 0.1451] | — | 51.5% |
| Ind1: CTQ-SF → Lack of premeditation → Addictive Features | 0.0047 | 0.0063 | [-0.0047, 0.0202] | — | — |
| Ind2: CTQ-SF → CDI → Addictive Features | 0.0262 | 0.0168 | [-0.0046, 0.0627] | — | — |
| Ind3: CTQ-SF → OSI Functions → Addictive Features | 0.0197 | 0.0191 | [-0.0125, 0.0620] | — | — |
| Ind4: CTQ-SF → Lack of premeditation → CDI → Addictive Features | 0.0017 | 0.0019 | [-0.0005, 0.0068] | — | — |
| Ind5: CTQ-SF → Lack of premeditation → OSI Functions → Addictive Features | 0.0002 | 0.0025 | [-0.0061, 0.0046] | — | — |
| Ind6: CTQ-SF → CDI → OSI Functions → Addictive Features | 0.0253 | 0.0100 | [0.0107, 0.0488] | — | 16.4% * |
| Ind7: CTQ-SF → Lack of premeditation → CDI → OSI Functions → Addictive Features | 0.0016 | 0.0013 | [-0.0004, 0.0046] | — | — |
| Lack of perseverance |  |  |  |  |  |
| Total effect (*c*) | 0.1543 | 0.0354 | [0.0844, 0.2242] | <0.001 | 100% |
| Direct effect (*c*′) | 0.0767 | 0.0334 | [0.0107, 0.1428] | 0.023 | — |
| Total indirect | 0.0775 | 0.0282 | [0.0278, 0.1390] | — | 50.2% |
| Ind1: CTQ-SF → Lack of perseverance → Addictive Features | -0.0005 | 0.0040 | [-0.0097, 0.0082] | — | — |
| Ind2: CTQ-SF → CDI → Addictive Features | 0.0299 | 0.0175 | [-0.0021, 0.0678] | — | — |
| Ind3: CTQ-SF → OSI Functions → Addictive Features | 0.0196 | 0.0192 | [-0.0126, 0.0635] | — | — |
| Ind4: CTQ-SF → Lack of perseverance → CDI → Addictive Features | 0.0012 | 0.0017 | [-0.0006, 0.0059] | — | — |
| Ind5: CTQ-SF → Lack of perseverance → OSI Functions → Addictive Features | 0.0012 | 0.0021 | [-0.0023, 0.0064] | — | — |
| Ind6: CTQ-SF → CDI → OSI Functions → Addictive Features | 0.0251 | 0.0095 | [0.0100, 0.0473] | — | 16.3% * |
| Ind7: CTQ-SF → Lack of perseverance → CDI → OSI Functions → Addictive Features | 0.0010 | 0.0011 | [-0.0005, 0.0039] | — | — |

Note. *N* = 163. Covariates: gender, age, years of education, total illness duration (months), number of MDD episodes, and parental marital status (two dummy variables, married as reference). In each model, the designated facet replaced the SUPPS-P total score as the first mediator (CTQ-SF → facet → CDI → OSI Functions → addiction); depression (CDI) and NSSI function (OSI Functions) remained the second and third mediators. 95% CIs for indirect effects are percentile bootstrap CIs based on 5,000 resamples; *p* values for the total and direct effects are from OLS *t*-tests. Proportion mediated = indirect effect / total effect; shown only for significant indirect effects (marked *), i.e., 95% bootstrap CI excluding zero.

**Supplementary Table S14. Exploratory facet-level serial mediation analyses: each SUPPS-P facet replacing the total score as the first mediator (Imputation_10; 5,000 bootstrap resamples)**

| **Facet / path** | **Effect (*B*)** | **Boot *SE*** | **95% CI** | ***p*** | **Proportion mediated** |
| --- | --- | --- | --- | --- | --- |
| Negative urgency |  |  |  |  |  |
| Total effect (*c*) | 0.1397 | 0.0356 | [0.0694, 0.2099] | <0.001 | 100% |
| Direct effect (*c*′) | 0.0583 | 0.0331 | [-0.0071, 0.1238] | 0.080 | — |
| Total indirect | 0.0813 | 0.0296 | [0.0316, 0.1467] | — | 58.2% |
| Ind1: CTQ-SF → Negative urgency → Addictive Features | 0.0029 | 0.0057 | [-0.0050, 0.0185] | — | — |
| Ind2: CTQ-SF → CDI → Addictive Features | 0.0332 | 0.0183 | [0.0001, 0.0731] | — | 23.8% * |
| Ind3: CTQ-SF → OSI Functions → Addictive Features | 0.0189 | 0.0163 | [-0.0080, 0.0561] | — | — |
| Ind4: CTQ-SF → Negative urgency → CDI → Addictive Features | 0.0007 | 0.0012 | [-0.0011, 0.0037] | — | — |
| Ind5: CTQ-SF → Negative urgency → OSI Functions → Addictive Features | 0.0029 | 0.0047 | [-0.0044, 0.0142] | — | — |
| Ind6: CTQ-SF → CDI → OSI Functions → Addictive Features | 0.0223 | 0.0091 | [0.0085, 0.0434] | — | 16.0% * |
| Ind7: CTQ-SF → Negative urgency → CDI → OSI Functions → Addictive Features | 0.0005 | 0.0008 | [-0.0008, 0.0024] | — | — |
| Positive urgency |  |  |  |  |  |
| Total effect (*c*) | 0.1397 | 0.0356 | [0.0694, 0.2099] | <0.001 | 100% |
| Direct effect (*c*′) | 0.0545 | 0.0332 | [-0.0111, 0.1201] | 0.103 | — |
| Total indirect | 0.0852 | 0.0300 | [0.0330, 0.1505] | — | 61.0% |
| Ind1: CTQ-SF → Positive urgency → Addictive Features | 0.0080 | 0.0084 | [-0.0044, 0.0282] | — | — |
| Ind2: CTQ-SF → CDI → Addictive Features | 0.0276 | 0.0151 | [-0.0012, 0.0591] | — | — |
| Ind3: CTQ-SF → OSI Functions → Addictive Features | 0.0154 | 0.0161 | [-0.0122, 0.0510] | — | — |
| Ind4: CTQ-SF → Positive urgency → CDI → Addictive Features | 0.0039 | 0.0036 | [-0.0002, 0.0133] | — | — |
| Ind5: CTQ-SF → Positive urgency → OSI Functions → Addictive Features | 0.0100 | 0.0063 | [0.0012, 0.0253] | — | 7.2% * |
| Ind6: CTQ-SF → CDI → OSI Functions → Addictive Features | 0.0178 | 0.0081 | [0.0059, 0.0377] | — | 12.7% * |
| Ind7: CTQ-SF → Positive urgency → CDI → OSI Functions → Addictive Features | 0.0025 | 0.0018 | [0.0003, 0.0071] | — | 1.8% * |
| Sensation seeking |  |  |  |  |  |
| Total effect (*c*) | 0.1397 | 0.0356 | [0.0694, 0.2099] | <0.001 | 100% |
| Direct effect (*c*′) | 0.0506 | 0.0337 | [-0.0160, 0.1172] | 0.136 | — |
| Total indirect | 0.0891 | 0.0303 | [0.0373, 0.1552] | — | 63.8% |
| Ind1: CTQ-SF → Sensation seeking → Addictive Features | 0.0069 | 0.0080 | [-0.0068, 0.0253] | — | — |
| Ind2: CTQ-SF → CDI → Addictive Features | 0.0329 | 0.0179 | [0.0011, 0.0705] | — | 23.6% * |
| Ind3: CTQ-SF → OSI Functions → Addictive Features | 0.0102 | 0.0173 | [-0.0192, 0.0502] | — | — |
| Ind4: CTQ-SF → Sensation seeking → CDI → Addictive Features | 0.0021 | 0.0022 | [-0.0012, 0.0073] | — | — |
| Ind5: CTQ-SF → Sensation seeking → OSI Functions → Addictive Features | 0.0107 | 0.0062 | [0.0015, 0.0252] | — | 7.7% * |
| Ind6: CTQ-SF → CDI → OSI Functions → Addictive Features | 0.0247 | 0.0094 | [0.0100, 0.0460] | — | 17.7% * |
| Ind7: CTQ-SF → Sensation seeking → CDI → OSI Functions → Addictive Features | 0.0016 | 0.0016 | [-0.0007, 0.0053] | — | — |
| Lack of premeditation |  |  |  |  |  |
| Total effect (*c*) | 0.1397 | 0.0356 | [0.0694, 0.2099] | <0.001 | 100% |
| Direct effect (*c*′) | 0.0538 | 0.0333 | [-0.0121, 0.1196] | 0.109 | — |
| Total indirect | 0.0859 | 0.0297 | [0.0355, 0.1522] | — | 61.5% |
| Ind1: CTQ-SF → Lack of premeditation → Addictive Features | 0.0041 | 0.0061 | [-0.0051, 0.0196] | — | — |
| Ind2: CTQ-SF → CDI → Addictive Features | 0.0310 | 0.0172 | [-0.0010, 0.0679] | — | — |
| Ind3: CTQ-SF → OSI Functions → Addictive Features | 0.0194 | 0.0194 | [-0.0123, 0.0636] | — | — |
| Ind4: CTQ-SF → Lack of premeditation → CDI → Addictive Features | 0.0019 | 0.0021 | [-0.0004, 0.0074] | — | — |
| Ind5: CTQ-SF → Lack of premeditation → OSI Functions → Addictive Features | 0.0001 | 0.0026 | [-0.0066, 0.0047] | — | — |
| Ind6: CTQ-SF → CDI → OSI Functions → Addictive Features | 0.0277 | 0.0106 | [0.0119, 0.0537] | — | 19.8% * |
| Ind7: CTQ-SF → Lack of premeditation → CDI → OSI Functions → Addictive Features | 0.0017 | 0.0013 | [-0.0004, 0.0048] | — | — |
| Lack of perseverance |  |  |  |  |  |
| Total effect (*c*) | 0.1397 | 0.0356 | [0.0694, 0.2099] | <0.001 | 100% |
| Direct effect (*c*′) | 0.0556 | 0.0333 | [-0.0103, 0.1214] | 0.098 | — |
| Total indirect | 0.0841 | 0.0295 | [0.0351, 0.1485] | — | 60.2% |
| Ind1: CTQ-SF → Lack of perseverance → Addictive Features | -0.0017 | 0.0041 | [-0.0110, 0.0060] | — | — |
| Ind2: CTQ-SF → CDI → Addictive Features | 0.0353 | 0.0182 | [0.0031, 0.0747] | — | 25.3% * |
| Ind3: CTQ-SF → OSI Functions → Addictive Features | 0.0193 | 0.0187 | [-0.0118, 0.0613] | — | — |
| Ind4: CTQ-SF → Lack of perseverance → CDI → Addictive Features | 0.0013 | 0.0017 | [-0.0007, 0.0060] | — | — |
| Ind5: CTQ-SF → Lack of perseverance → OSI Functions → Addictive Features | 0.0013 | 0.0021 | [-0.0023, 0.0066] | — | — |
| Ind6: CTQ-SF → CDI → OSI Functions → Addictive Features | 0.0276 | 0.0105 | [0.0116, 0.0530] | — | 19.8% * |
| Ind7: CTQ-SF → Lack of perseverance → CDI → OSI Functions → Addictive Features | 0.0010 | 0.0012 | [-0.0006, 0.0040] | — | — |

Note. *N* = 163. Covariates: gender, age, years of education, total illness duration (months), number of MDD episodes, and parental marital status (two dummy variables, married as reference). In each model, the designated facet replaced the SUPPS-P total score as the first mediator (CTQ-SF → facet → CDI → OSI Functions → addiction); depression (CDI) and NSSI function (OSI Functions) remained the second and third mediators. 95% CIs for indirect effects are percentile bootstrap CIs based on 5,000 resamples; *p* values for the total and direct effects are from OLS *t*-tests. Proportion mediated = indirect effect / total effect; shown only for significant indirect effects (marked *), i.e., 95% bootstrap CI excluding zero.

**Supplementary Table S15. Sensitivity analysis: serial mediation with the order of the first two mediators reversed (childhood trauma → depression → impulsivity → NSSI function → NSSI addiction; Imputation_1; 5,000 bootstrap resamples)**

| **Path** | **Effect (*B*)** | **Boot *SE*** | **95% CI** | ***p*** | **Proportion mediated** |
| --- | --- | --- | --- | --- | --- |
| Total effect (*c*) | 0.1513 | 0.0354 | [0.0814, 0.2212] | <0.001 | 100% |
| Direct effect (*c*′) | 0.0662 | 0.0338 | [-0.0006, 0.1330] | 0.052 | — |
| Total indirect | 0.0851 | 0.0290 | [0.0369, 0.1509] | — | 56.2% * |
| Ind1: CTQ-SF → CDI → Addictive Features | 0.0284 | 0.0179 | [-0.0040, 0.0661] | — | — |
| Ind2: CTQ-SF → SUPPS-P → Addictive Features | 0.0095 | 0.0088 | [-0.0021, 0.0314] | — | — |
| Ind3: CTQ-SF → OSI Functions → Addictive Features | 0.0066 | 0.0129 | [-0.0170, 0.0352] | — | — |
| Ind4: CTQ-SF → CDI → SUPPS-P → Addictive Features | 0.0091 | 0.0055 | [0.0003, 0.0213] | — | 6.0% * |
| Ind5: CTQ-SF → CDI → OSI Functions → Addictive Features | 0.0168 | 0.0080 | [0.0050, 0.0355] | — | 11.1% * |
| Ind6: CTQ-SF → SUPPS-P → OSI Functions → Addictive Features | 0.0075 | 0.0061 | [-0.0016, 0.0226] | — | — |
| Ind7: CTQ-SF → CDI → SUPPS-P → OSI Functions → Addictive Features | 0.0072 | 0.0037 | [0.0021, 0.0164] | — | 4.8% * |

Note. *N* = 163. Covariates: gender, age, years of education, total illness duration (months), number of MDD episodes, and parental marital status (two dummy variables, married as reference). Mediators entered as CDI (M1), SUPPS-P (M2), and OSI Functions (M3), i.e., depression preceding impulsivity. 95% CIs for indirect effects are percentile bootstrap CIs based on 5,000 resamples; *p* values for the total and direct effects are from OLS *t*-tests. Proportion mediated = indirect effect / total effect; shown only for significant indirect effects (marked *), i.e., 95% bootstrap CI excluding zero. The total and direct effects are identical to those of the primary model because the outcome equation is saturated with respect to the mediator set.

**Supplementary Table S16. Sensitivity analysis: serial mediation with the order of the first two mediators reversed (childhood trauma → depression → impulsivity → NSSI function → NSSI addiction; Imputation_5; 5,000 bootstrap resamples)**

| **Path** | **Effect (*B*)** | **Boot *SE*** | **95% CI** | ***p*** | **Proportion mediated** |
| --- | --- | --- | --- | --- | --- |
| Total effect (*c*) | 0.1473 | 0.0353 | [0.0775, 0.2170] | <0.001 | 100% |
| Direct effect (*c*′) | 0.0640 | 0.0334 | [-0.0019, 0.1299] | 0.057 | — |
| Total indirect | 0.0832 | 0.0297 | [0.0330, 0.1514] | — | 56.5% * |
| Ind1: CTQ-SF → CDI → Addictive Features | 0.0273 | 0.0179 | [-0.0048, 0.0657] | — | — |
| Ind2: CTQ-SF → SUPPS-P → Addictive Features | 0.0069 | 0.0077 | [-0.0029, 0.0268] | — | — |
| Ind3: CTQ-SF → OSI Functions → Addictive Features | 0.0091 | 0.0147 | [-0.0174, 0.0412] | — | — |
| Ind4: CTQ-SF → CDI → SUPPS-P → Addictive Features | 0.0079 | 0.0052 | [-0.0006, 0.0199] | — | — |
| Ind5: CTQ-SF → CDI → OSI Functions → Addictive Features | 0.0178 | 0.0082 | [0.0062, 0.0376] | — | 12.1% * |
| Ind6: CTQ-SF → SUPPS-P → OSI Functions → Addictive Features | 0.0067 | 0.0062 | [-0.0027, 0.0221] | — | — |
| Ind7: CTQ-SF → CDI → SUPPS-P → OSI Functions → Addictive Features | 0.0076 | 0.0038 | [0.0023, 0.0169] | — | 5.2% * |

Note. *N* = 163. Covariates: gender, age, years of education, total illness duration (months), number of MDD episodes, and parental marital status (two dummy variables, married as reference). Mediators entered as CDI (M1), SUPPS-P (M2), and OSI Functions (M3), i.e., depression preceding impulsivity. 95% CIs for indirect effects are percentile bootstrap CIs based on 5,000 resamples; *p* values for the total and direct effects are from OLS *t*-tests. Proportion mediated = indirect effect / total effect; shown only for significant indirect effects (marked *), i.e., 95% bootstrap CI excluding zero. The total and direct effects are identical to those of the primary model because the outcome equation is saturated with respect to the mediator set.

**Supplementary Table S17. Sensitivity analysis: serial mediation with the order of the first two mediators reversed (childhood trauma → depression → impulsivity → NSSI function → NSSI addiction; Imputation_10; 5,000 bootstrap resamples)**

| **Path** | **Effect (*B*)** | **Boot *SE*** | **95% CI** | ***p*** | **Proportion mediated** |
| --- | --- | --- | --- | --- | --- |
| Total effect (*c*) | 0.1460 | 0.0352 | [0.0765, 0.2155] | <0.001 | 100% |
| Direct effect (*c*′) | 0.0716 | 0.0330 | [0.0065, 0.1368] | 0.031 | — |
| Total indirect | 0.0744 | 0.0295 | [0.0252, 0.1430] | — | 51.0% * |
| Ind1: CTQ-SF → CDI → Addictive Features | 0.0269 | 0.0167 | [-0.0041, 0.0617] | — | — |
| Ind2: CTQ-SF → SUPPS-P → Addictive Features | 0.0070 | 0.0081 | [-0.0034, 0.0282] | — | — |
| Ind3: CTQ-SF → OSI Functions → Addictive Features | 0.0035 | 0.0151 | [-0.0232, 0.0370] | — | — |
| Ind4: CTQ-SF → CDI → SUPPS-P → Addictive Features | 0.0078 | 0.0049 | [-0.0001, 0.0191] | — | — |
| Ind5: CTQ-SF → CDI → OSI Functions → Addictive Features | 0.0173 | 0.0079 | [0.0054, 0.0361] | — | 11.8% * |
| Ind6: CTQ-SF → SUPPS-P → OSI Functions → Addictive Features | 0.0056 | 0.0055 | [-0.0028, 0.0194] | — | — |
| Ind7: CTQ-SF → CDI → SUPPS-P → OSI Functions → Addictive Features | 0.0062 | 0.0034 | [0.0017, 0.0148] | — | 4.2% * |

Note. *N* = 163. Covariates: gender, age, years of education, total illness duration (months), number of MDD episodes, and parental marital status (two dummy variables, married as reference). Mediators entered as CDI (M1), SUPPS-P (M2), and OSI Functions (M3), i.e., depression preceding impulsivity. 95% CIs for indirect effects are percentile bootstrap CIs based on 5,000 resamples; *p* values for the total and direct effects are from OLS *t*-tests. Proportion mediated = indirect effect / total effect; shown only for significant indirect effects (marked *), i.e., 95% bootstrap CI excluding zero. The total and direct effects are identical to those of the primary model because the outcome equation is saturated with respect to the mediator set.
